# Teleost swim bladder, an ancient air-filled organ that elicits mucosal immune responses

**DOI:** 10.1038/s41421-022-00393-3

**Published:** 2022-04-05

**Authors:** Yongyao Yu, Zhenyu Huang, Weiguang Kong, Fen Dong, Xiaoting Zhang, Xue Zhai, Gaofeng Cheng, Mengting Zhan, Jiafeng Cao, Liguo Ding, Guangkun Han, Fumio Takizawa, Yang Ding, J. Oriol Sunyer, Zhen Xu

**Affiliations:** 1 Department of Aquatic Animal Medicine, College of Fisheries, Huazhong Agricultural University, Wuhan, Hubei China; 2 State Key Laboratory of Freshwater Ecology and Biotechnology, Institute of Hydrobiology, Chinese Academy of Sciences, Wuhan, Hubei China; 3 Faculty of Marine Science and Technology, Fukui Prefectural University, Obama, Fukui, Japan; 4 Department of Pathobiology, School of Veterinary Medicine, University of Pennsylvania, Philadelphia, Pennsylvania, PA USA; 5 Laboratory for Marine Biology and Biotechnology, Qingdao National Laboratory for Marine Science and Technology, Qingdao, Shandong China

**Keywords:** Immunology, Innate immunity

## Abstract

The air-filled organs (AOs) of vertebrates (lungs and swim bladders) have evolved unique functions (air-breathing or buoyancy control in water) to adapt to different environments. Thus far, immune responses to microbes in AOs have been described exclusively in the lungs of tetrapods. Similar to lungs, swim bladders (SBs) represent a mucosal surface, a feature that leads us to hypothesize a role for SB in immunity. In this study, we demonstrate that secretory IgT (sIgT) is the key SB immunoglobulin (Ig) responding to the viral challenge, and the only Ig involved in viral neutralization in that organ. In support of these findings, we found that the viral load of the SB from fish devoid of sIgT was much higher than that of control fish. Interestingly, similar to the lungs in mammals, the SB represents the mucosal surface in fish with the lowest content of microbiota. Moreover, sIgT is the main Ig class found coating their surface, suggesting a key role of this Ig in the homeostasis of the SB microbiota. In addition to the well-established role of SB in buoyancy control, our findings reveal a previously unrecognized function of teleost SB in adaptive mucosal immune responses upon pathogenic challenge, as well as a previously unidentified role of sIgT in antiviral defense. Overall, our findings indicate that despite the phylogenetic distance and physiological roles of teleost SB and mammalian lungs, they both have evolved analogous mucosal immune responses against microbes which likely originated independently through a process of convergent evolution.

## Introduction

Air-filled organs (AOs) emerged ~400 million years ago in early ray-finned fishes (Actinopterygii) and are a defining and crucial feature for the survival of bony vertebrates^1–3^. Throughout the evolution of bony vertebrates, AOs underwent important adaptive changes in response to different environmental pressures, particularly during the water-to-land transition in the Devonian^4^. Interestingly, although the common ancestor of AOs can be traced back to early ray-finned fishes, which featured primitive lungs, teleost fish evolved swim bladders (SBs) which play a key role in buoyancy control, although in some species SBs can also have auxiliary functions in respiration, sound production, and hearing^5,6^. In contrast, when vertebrates colonized terrestrial ecosystems, basal lobe-finned fishes (e.g., lungfish) evolved lungs that were functionally similar to those in tetrapods to serve as gas exchange organs, thus enabling them to breathe air^4,7,8^. Recently, increasing evidence from morphological, phylogenetic, and genetic data has confirmed the evolutionary relationships between the lungs and SB^4,9,10^. Both organs originated most likely from primitive lungs in the last common ancestor of early ray-finned fish^4^. It is also worth noting that both the lungs and SB developed from the anterior foregut endoderm, albeit following different budding patterns, that is, lungs bud ventrally and SB bud dorsally^10^.

The lungs are constantly exposed to the environment and are therefore at risk of being infected with pathogens such as influenza and SARS-CoV-2 viruses^11,12^. To fight pathogens, the lungs have evolved type-I mucosal epithelia and inducible mucosal-associated lymphoid tissue (MALT)^13–15^. Critically, the secretory IgA (sIgA) locally induced in the lungs MALT are transported by the polymeric immunoglobulin receptor (pIgR) to the mucosa surface for the elimination of respiratory antigens or neutralization of respiratory viruses^16–19^. Teleost fish represent the oldest bony vertebrates featuring MALT and bonafide immunoglobulins (Igs)^20–22^. Breaking the old paradigm that mucosal Igs were present only in tetrapod species, we have previously shown that teleosts contain IgT, the most ancient Ig specialized in mucosal immunity against parasitic and bacterial pathogens^23–25^. Moreover, we have demonstrated that, analogously to mammalian IgA, teleost secretory IgT (sIgT) is the main sIg isotype coating the microbiota of mucosal surfaces. The crucial role of sIgT in the control of mucosal pathogens and microbiota was recently confirmed by our groups using fish devoid of IgT. Depletion of IgT^+^ B-cells in these animals induced severe dysbiosis and turned them significantly more susceptible to a mucosal pathogen^26,27^.

While the lungs of tetrapods are known to contain MALT, which is critical in eliminating pathogens, very little is known about the evolutionary origins of AOs MALT in non-tetrapods and its primordial roles in immune defense and microbiota homeostasis. Given the mucosal nature of the SB surface and the common evolutionary ancestry between lungs and SB, we hypothesized that AOs in both primitive and modern bony vertebrates must have evolved analogous molecular mechanisms for combating microbes. This study was therefore undertaken to examine whether the AOs of an aquatic species (i.e., teleost SB) could fulfill immune roles. Thus, in addition to its unique role in buoyancy control, we set to investigate whether the SB might also represent an ancient mucosal immune organ.

Supporting the aforementioned hypothesis, here we show that teleost SB possesses a bonafide MALT with striking structural and functional immune commonalities with that of lung and other type-I mucosal surfaces. We found that infection of the SB with a virus elicited a strong innate immune response in the SB, and that reinfection induced local IgT^+^ B-cells proliferation in the SB as well as virus-specific sIgT. Importantly, the induced sIgT was able to neutralize the virus thus exposing a previously unrecognized effector function of sIgT in viral defense. Reinforcing the role of sIgT in SB immunity and viral control, we found that the SB of fish devoid of IgT was significantly more susceptible to the virus than that of IgT-containing fish. Here we also show that, the SB contains a microbiota population that is prevalently coated by sIgT, suggesting a role for sIgT in the control of SB microbiota.

In addition to its well-known role in buoyancy control, our results reveal a previously unrecognized role of teleost SB in immunity thus providing insight into the evolution of immune responses to pathogens and microbiota in an ancient AO. Moreover, as zebrafish has been used, through infection of their SB as model species to study several human lung pathogens^28–30^ (including SARS-CoV-2), our findings will also be instrumental in furthering the use of teleost fish as animal models to understand host–pathogen interactions for a number of respiratory pathogens, as well as the outcomes of therapeutic interventions against them, and the associated side effects.

## Results

### The teleost SB contains a MALT

**Fig. 1:**
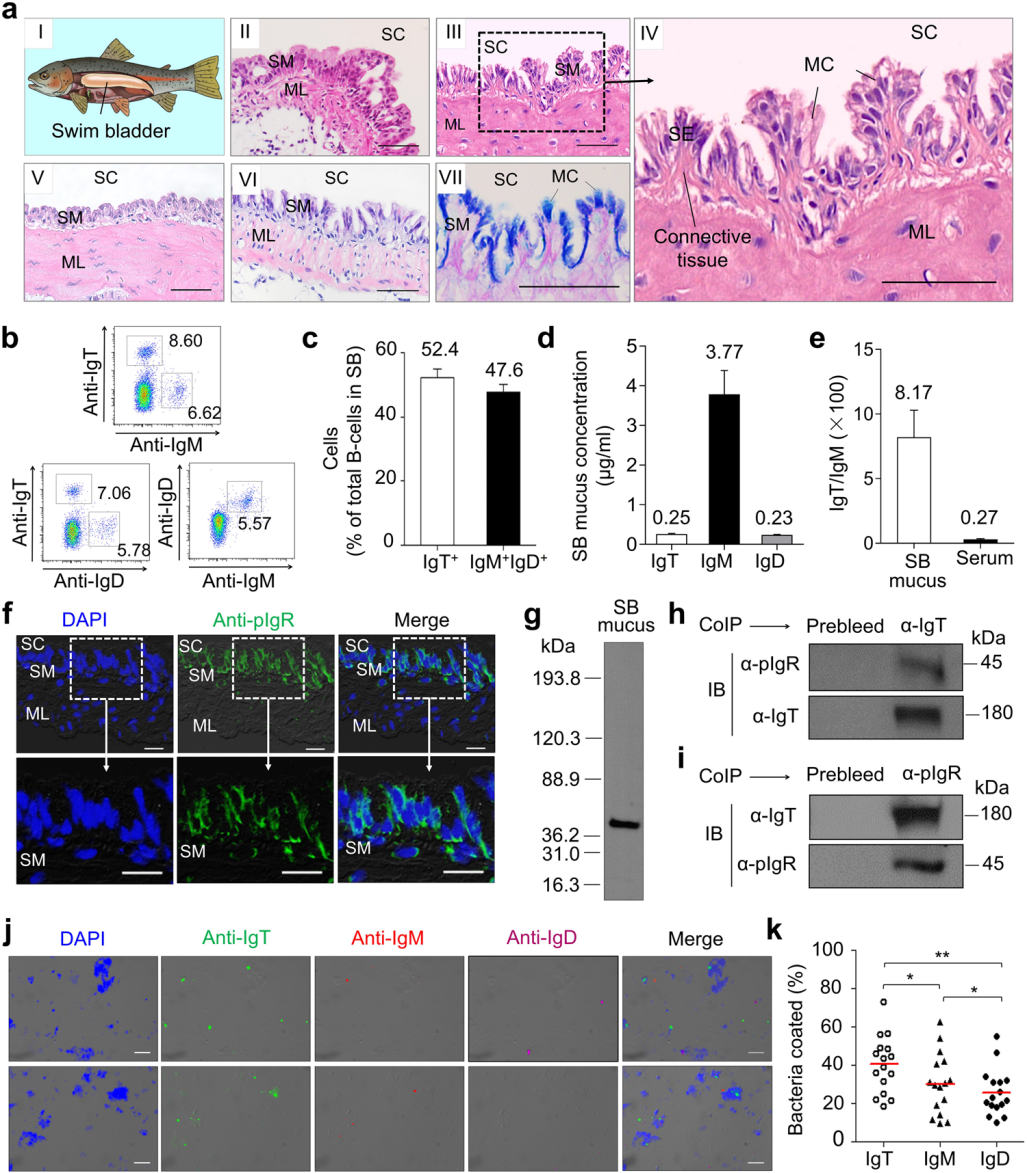
Morphological structure and presence of MALT in the swim bladder (SB) mucosa of trout. **a** The anatomy (**a I**) and the structure (**a II**–**a VII**) of teleost SB. H&E staining of SB mucosa obtained from four different teleost species, including *Danio rerio* (**a II**), *Oncorhynchus mykiss* (**a III**; **a IV**, enlarged section of the area outlined in **III**), *Osteoglossum bicirrhosum* (**a V**) and *Salmo salar* (**a VI**), and AB-PAS stain of the SB mucosa in naive *Oncorhynchus mykiss* (**a VII**). SC SB cavity, SE SB epithelium, SM SB mucosa, MC mucus-producing cells, ML muscle layer. Scale bars, 50 μm. **b** Representative flow cytometry staining of leukocytes from SB with anti-trout Igs mAbs. Numbers adjacent to outlined areas of dot-plots indicate percentage of different B-cell populations (e.g., IgT^+^ B-cells, IgM^+^ B-cells, IgD^+^ B-cells, and IgM^+^IgD^+^ B-cells) in the lymphocyte gate. **c** Frequency of IgT^+^ and IgM^+^IgD^+^ B-cells among the total B-cells (*n* = 22). **d** Concentration of IgT, IgM, and IgD protein levels in SB mucus (*n* = 13). **e** Ratio of IgT to IgM concentration in SB mucus and serum, calculated from the values shown in **d** and Supplementary Fig. S1d. **f** Immunofluorescence staining for trout pIgR in paraffin-sections of trout SB mucosa. Differential interference contrast (DIC) images of SB mucosa paraffin-sections were stained with DAPI (blue) for nuclei and anti-trout pIgR pAb (green) (*n* = 9) (Isotype-matched control antibody staining is shown in Supplementary Fig. S2a). SC SB cavity, SM SB mucosa, ML muscle layer. Scale bars, 20 μm. **g** SDS-PAGE under reducing conditions of trout SB mucus, followed by immunoblot (IB) analysis using anti-trout pIgR pAb. **h** Co-immunoprecipitation (CoIP) of SB mucus with anti-trout IgT-specific pAb, followed by IB under reducing conditions (pIgR detection, upper panels) or non-reducing conditions (IgT detection, lower panels). **i** CoIP of SB mucus with anti-trout pIgR pAb, followed by IB under non-reducing conditions (IgT detection, upper panels) or reducing conditions (pIgR detection, lower panels). IgG purified from rabbit’s serum before immunization (Prebleed) served as negative control for rabbit anti-trout IgT and rabbit anti-trout pIgR pAb, respectively (left lane on each panel for **h** and **i**). **j** DIC images of SB bacteria stained with a DAPI (blue), anti-IgT (green), anti-IgM (red), or anti-IgD (magenta), and merging IgT, IgM, and IgD staining (Merge) (Isotype-matched control antibody staining is shown in Supplementary Fig. S3b). Scale bars, 5 μm. **k** Percentages of SB bacteria coated with IgT, IgM, or IgD analyzed by flow cytometry (*n* = 16). The median percentage is shown by a red line. Statistical differences were evaluated by one-way ANOVA with Bonferroni correction. Data in **k** are representative of at least three independent experiments (mean ± SEM). ANOVA, analysis of variance. *P < 0.05, and **P < 0.01.

The teleost SB is an internal gas-filled organ located in the dorsal portion of the body (Fig. 1a i; Supplementary Fig. S1a). This organ is comprised of a thin, flexible membrane with very few blood vessels, which contracts or expands according to the ambient pressure (Supplementary Fig. S1a)^31^. To identify whether teleost SB contains diffuse lymphoid cells or lymphoid tissue similar to that of other teleost MALTs, we first examined its histological organization via hematoxylin/eosin (H&E) staining of teleost SB tissue obtained from three different teleost families (Cyprinidae (order Cypriniforms), Salmonidae (order Salmoniforms), and Osteoglossidae (order Osteoglossiforms)). We found that SB cavity (SC) is covered by a mucosa (SB mucosa [SM]) and a muscle layer (ML). The SM of all species studied had a similar overall structure, including that of zebrafish (*Danio rerio*) (Fig. 1a ii), rainbow trout (*Oncorhynchus mykiss*) (Fig. 1a iii, iv), silver arowana (*Osteoglossum bicirrhosum*) (Fig. 1a v), and Atlantic salmon (*Salmo salar*) (Fig. 1a vi). As shown in the rainbow trout enlarged SB structure (Fig. 1a iv), SM had two main layers, an epithelium layer (SE) and an underlying layer of connective tissue. Alcian Blue-Periodic acid-Schiff (AB-PAS) analysis identified a large number of mucus-producing cells (MC) in the SE of the SB in rainbow trout (Fig. 1a vii). Our subsequent analyses focused on the SB of rainbow trout, our model species. With specific monoclonal antibodies (mAbs) to trout Igs (anti-trout IgT, IgM, and IgD), flow cytometry analysis showed that two main B-cell subsets were present in SB tissue (Fig. 1b), one expressing only surface IgT (IgT^+^ B-cells), and the second one expressing both surfaces IgM and IgD (IgM^+^IgD^+^ B-cells). Among these two populations, IgT^+^ B-cells were the most abundant subset (52.4% of total B-cells) (Fig. 1c), as shown also for all other trout MALTs analyzed thus far^22^.

To characterize the presence and structure of trout Igs in SB mucus, we first collected, processed, and loaded the SB mucus of rainbow trout into a gel filtration column. A large portion of the IgT in the SB mucus was in polymeric form, as it eluted at a fraction similar to that of trout tetrameric IgM (Supplementary Fig. S1b). SDS-PAGE under non-reducing conditions showed that polymeric IgT in SB mucus migrated to the same position as monomeric IgT, indicating that IgT subunits were associated via noncovalent interactions (Supplementary Fig. S1c, right panel). Next, we measured the protein levels of the three Igs in SB mucus and serum of rainbow trout (Fig. 1d; Supplementary Fig. S1d) and found that although both IgT and IgD protein levels were lower than those of IgM, the IgT/IgM and IgD/IgM ratios in the SB mucus were ~30- and 6-fold higher compared with those in serum, respectively (Fig. 1e; Supplementary Fig. S1e). This result was consistent with the observations in other teleost MALTs^22^. The polymeric Ig receptor (pIgR) mediates the transport of mucosal Ig across the epithelium in teleost and other vertebrate MALTs^23–26,32–35^. Immunofluorescence and western blot analyses indicated that a large number of pIgR-containing cells were located in the trout SB epithelium (Fig. 1f; isotype-matched control antibodies as shown in Supplementary Fig. S2a), whereas the trout secretory component (tSC) fragment of trout pIgR was present in the SB mucus (Fig. 1g). Using co-immunoprecipitation assays, we found that antibodies against trout IgT could co-immunoprecipitate tSC in the SB mucus (Fig. 1h), and sIgT in SB mucus could also be immunoprecipitated by anti-trout pIgR antibody (Fig. 1i), indicating an interaction between sIgT and pIgR in the SB mucosa.

A previous study characterized the presence of microbiota on the trout SB^36^. By qPCR, we found that SB had the lowest content of bacteria among all trout mucosal tissues identified thus far (SB, skin, stomach, pharynx, mouth, gill, and gut) (Supplementary Fig. S3a). Using immunofluorescence microscopy (Fig. 1j; isotype-matched control antibodies as shown Supplementary Fig. S3b) and flow cytometry (Fig. 1k; Supplementary Fig. S3c), we found that the SB microbiota were coated primarily by IgT (~40% of coating), and to a lesser extent by IgM and IgD (~30% and ~26% of coating respectively). Moreover, the immunoblotting analysis further demonstrated the percentage of three Igs coating on bacteria in total Igs concentration in SB mucus. Notably, we found that more than 36.5% of the total IgT present in SB mucus was used for bacterial coating, whereas only 19.4% of IgM and 11.1% of IgD were utilized (Supplementary Fig. S3d, e). Taken together, these results suggest that the SB of teleost fish contains a bonafide MALT in which sIgT and IgT^+^ B-cells are the dominant sIg and B cells respectively. Moreover, these data suggest that SB sIgT plays a role in the control of the microbiota.

### Establishment of a viral infection model in trout SB

**Fig. 2:**
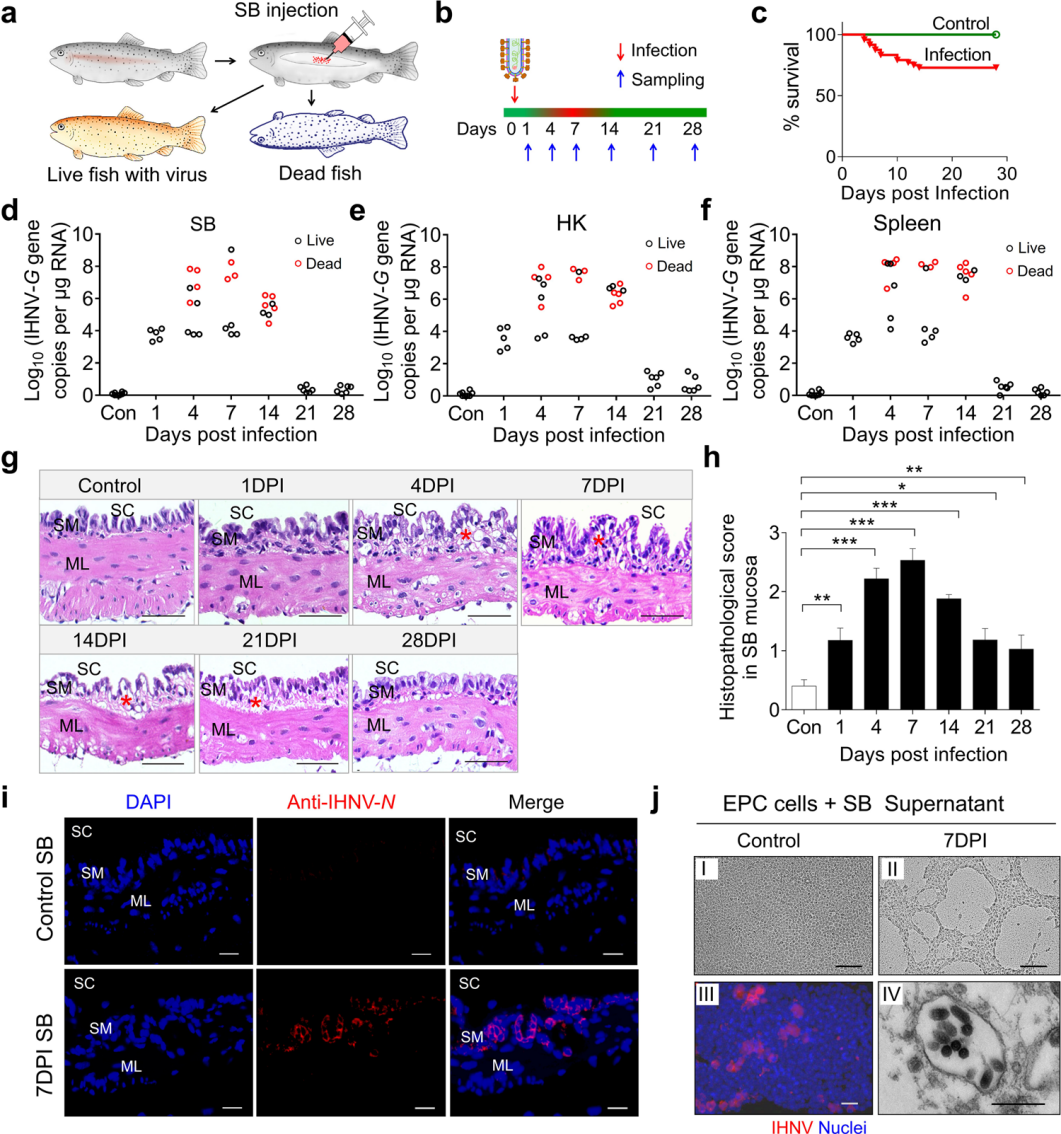
Infection model of SB with IHNV. **a**, **b** Trout were injected with 25 μL (1 × 10^5^ TCID_50_) of virus solution via intra-SB injection, and subsequently sacrificed at 1, 4, 7, 14, 21, and 28 DPI for tissue sample collection. The red color on the timeline represents dead fish or diseased fish which have symptoms of viral infection (i.e., slow swimming speed and inactive feeding when compared to control fish) while the green color represents normal behavior of a healthy fish**. c** Cumulative survival of control and IHNV-infected fish. **d**–**f** IHNV-*G* gene copies (Log_10_) were quantified using qPCR in fish tissues collected at 1, 4, 7, 14, 21, and 28 DPI. The virus was detected in the fish SB (**d**), head kidney (HK) (**e**), and spleen (**f**). Each circle represents one fish; red circles represent dead fish while black circles represent live fish. **g** Histological examination of SB from control fish and trout infected with IHNV at 1, 4, 7, 14, 21, and 28 DPI (*n* = 5–9). Red asterisks indicate the damage in SM. SC SB cavity, SM SB mucosa, ML muscle layer. Scale bars, 50 μm. **h** Pathology score of SB mucosa from control and infected fish. **i** Immunofluorescence staining of IHNV in SB paraffin-sections from control and 7 days infected fish (*n* = 5–11). IHNV (red) is stained with an anti-IHNV-*N* mAb; nuclei were stained with DAPI (blue). SC SB cavity, SM SB mucosa, ML muscle layer. Scale bars, 20 μm. **j** Cytopathic effect of IHNV on epithelioma papulosum cyprini (EPC) cells after culture with the supernatant of SB homogenates from control (**j** **I**) and the 7-day infected fish (**j II**). Immunofluorescence staining of IHNV using the anti-IHNV-*N* mAb, nuclei were stained with DAPI (blue) (**j III**). Electron microscope (EM) analysis of viral particles within EPC cells (**j IV**) from **j II**. Scale bars, 100 μm in **j I** and **j II**, 20 μm in **j III**, 500 nm in **j IV**. Statistical differences were evaluated by unpaired Student’s *t*-test. Data in **h** are representative of at least three independent experiments (mean ± SEM). *P < 0.05, **P < 0.01, and ***P < 0.001.

To assess whether the SB functions as an immune-responsive organ, we developed a viral infection model with infectious hematopoietic necrosis virus (IHNV) in which the virus was directly inoculated into the SB of live fish. As in Supplementary Fig. S4, we show that the medium can be accurately delivered into the SB lumen without affecting the swimming behavior of trout and its buoyancy control (Supplementary Video S1). Previous studies have shown that while IHNV targets the head kidney (HK) and spleen, it can also elicit a local mucosal immune response upon local infection^37^. Here we delivered IHNV (25 μL, 1 × 10^5^ TCID_50_ (median tissue culture infective dose)) via intra-SB cavity injection to directly infect the SB mucosa (Fig. 2a; Supplementary Fig. S4a). Upon infection, ~30% of the fish died within the first two weeks (Fig. 2b, c). Overall, the dead fish harbored a higher viral load in all analyzed organs (SB, HK, and spleen) when compared to live infected fish (Fig. 2d–f). Moreover, histological examination revealed significant histopathological changes in the SB mucosal epithelium of infected fish, especially at 7 days post-infection (DPI) (Fig. 2g, h). Moreover, significant diffuse tissue damage was also observed in the HK and spleen of infected fish compared with the control group (Supplementary Fig. S5a, b). At 4–7 DPI, high IHNV loads were observed in diseased trout SB, HK, spleen, eye, liver, and gills, while distant mucosal tissue (skin) contained a very low viral load (Supplementary Fig. S5c). Further, using an anti-IHNV-*N* mAb^38^ and immunofluorescence microscopy, IHNV was widely present in the SB epithelium of infected fish but not in that of control fish (Fig. 2i; isotype-matched control antibodies as shown in Supplementary Fig. S2b). At 7 DPI, the supernatant of SB homogenates was collected, and used to infect EPC cells, which resulted in cytopathic effects (CPE) of the cell cultures (Fig. 2j i, ii). IHNV was also detected using the anti-IHNV-*N* protein mAb (red) (Fig. 2j iii) and electron microscopy (Fig. 2j iv). Together, our results confirmed that our IHNV delivery strategy was successful in establishing an infection in the SB.

Next, we designed an experiment to test if trout that survived IHNV infection developed resistance to reinfection. To this end, control (fish never exposed to IHNV) and survivor (fish exposed three times to IHNV) groups were challenged at 75 DPI using a viral dose (25 μL, 1 × 10^6^ TCID_50_) via the same route of infection as described above, and fish were thereafter observed for 14 days (Supplementary Fig. S6a). Upon challenge, only ~30% of the trout in the control-challenge group survived, whereas all trout in the survivor-challenge group survived the high viral doses challenge (Supplementary Fig. S6b). We then repeated this experiment with the goal to sample fish at day 7 post-challenge. To this end, fish from control, control-challenge, 75 DPI survivor (75 DPI-S), and 75 DPI-S-challenge groups were sampled at 7 days post-challenge to evaluate viral loads in SB tissue using qPCR and immunofluorescence. Our qPCR data indicated that the viral load increased significantly in the control-challenge group compared with the control group. In contrast, a limited viral load was detected in the SB tissue of the 75 DPI-S-challenge group (Supplementary Fig. S6c). Histological examination indicated that significant signs of tissue damage were present in the SB mucosa epithelium of the control-challenge fish when compared to those of control fish (Supplementary Fig. S6d). In contrast, no visible histological changes were identified between the control, 75DPI-S, and 75DPI-S-challenge fish (Supplementary Fig. S6d, e). Using fluorescence microscopy, we detected that the virus was widely present in SB mucosa of the control-challenge fish while it was much less present in the control, 75DPI-S and 75DPI-S-challenge group (Supplementary Fig. S6f, g). Together, these data indicated that a strong protective immune response had been induced after three-time exposures of fish to IHNV.

### Innate and adaptive immune genes are induced in SB upon viral infection

**Fig. 3:**
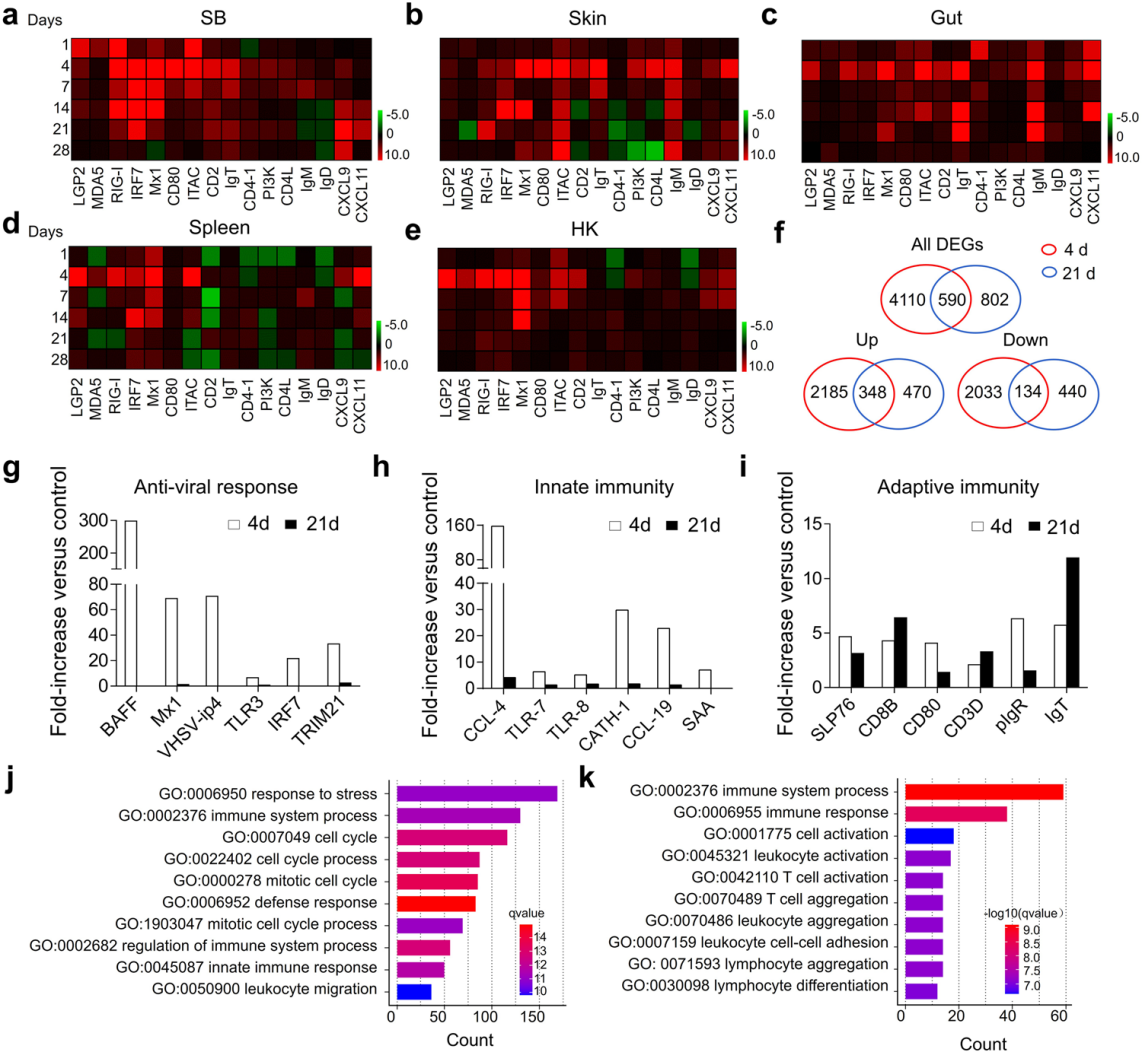
Expression of immune genes and transcriptome analyses of trout SB mucosa upon IHNV infection. **a**–**e** Heatmaps illustrate results from quantitative real-time PCR of transcripts for selected immune markers from several lymphoid organs of IHNV-infected versus control fish measured at 1, 4, 7, 14, 21, and 28 DPI, SB (**a**), skin (**b**), gut (**c**), spleen (**d**), and HK (**e**) (*n* = 9). Data are expressed as mean fold increase in expression. Color value: log_2_ (fold change). **f** Venn diagrams of RNA-Seq experiments showing the overlap of genes upregulated or downregulated in the SB mucosa of rainbow trout at 4 or 21 DPI versus control fish (*n* = 9). **g**–**i** Representative antiviral response (**g**), innate (**h**), and adaptive (**i**) immune genes modulated by IHNV infection at 4 or 21 DPI (*n* = 9). Data are expressed as mean fold increase in expression. **j**, **k** Biological processes that were significantly altered in SB at 4 (**j**) and 21 (**k**) DPI vs control fish revealed by RNA-Seq studies.

Next, qPCR was conducted to measure the expression of 16 immune-related genes and cell markers in the SB, skin, gut, spleen, and HK of trout at 1, 4, 7, 14, 21, and 28 days after IHNV infection (qPCR primer sequences are shown in Supplementary Table S1). A preliminary analysis suggested that upon infection, inflammation and strong innate and adaptive immune responses occurred in SB tissue in addition to the skin, gut, spleen, and HK tissues (Fig. 3a–e). Moreover, days 4 and 21 were the most relevant in terms of the intensity of the immune response (Fig. 3a–e), and therefore these two time points were selected for RNA-Seq analysis to further characterize the immune responses that took place in the SB tissue after IHNV infection. A total of 4700 (day 4) and 1392 (day 21) genes were significantly dysregulated following IHNV infection, of which 2533 and 818 genes were upregulated, whereas 2167 and 574 genes were downregulated at days 4 and 21, respectively (Fig. 3f). Among these differentially expressed genes (DEGs), we found a significant modification in the expression of genes involved in the antiviral activity (Fig. 3g), innate immunity (Fig. 3h), and adaptive immunity (Fig. 3i) both on days 4 and 21, indicating that IHNV infection could induce an intense innate immune response, in addition to adaptive immune responses. More importantly, we show increases in the mRNA expression levels of B cell (i.e., IgT, IgM, and IgD), T helper cell (i.e., CD4-1, CD4-2, and TCRα), and macrophage (i.e., MHC-II, MPEG1, and CSF1R) markers in trout SB upon infection with IHNV (Supplementary Fig. S7). Gene ontology (GO) enrichment analysis was then conducted to determine the biological processes in trout SB that were affected after IHNV infection. Interestingly, we found that the DEGs were primarily enriched in pathways associated with “cell cycle”, “cell adhesion”, and “immune system process”, all of which were involved in the innate and adaptive immune responses on days 4 and 21, respectively (Fig. 3j, k). Together, these data suggested that the SB functions as an immune-responsive organ.

### Viral-specific Ig and B-cell responses in trout SB

**Fig. 4:**
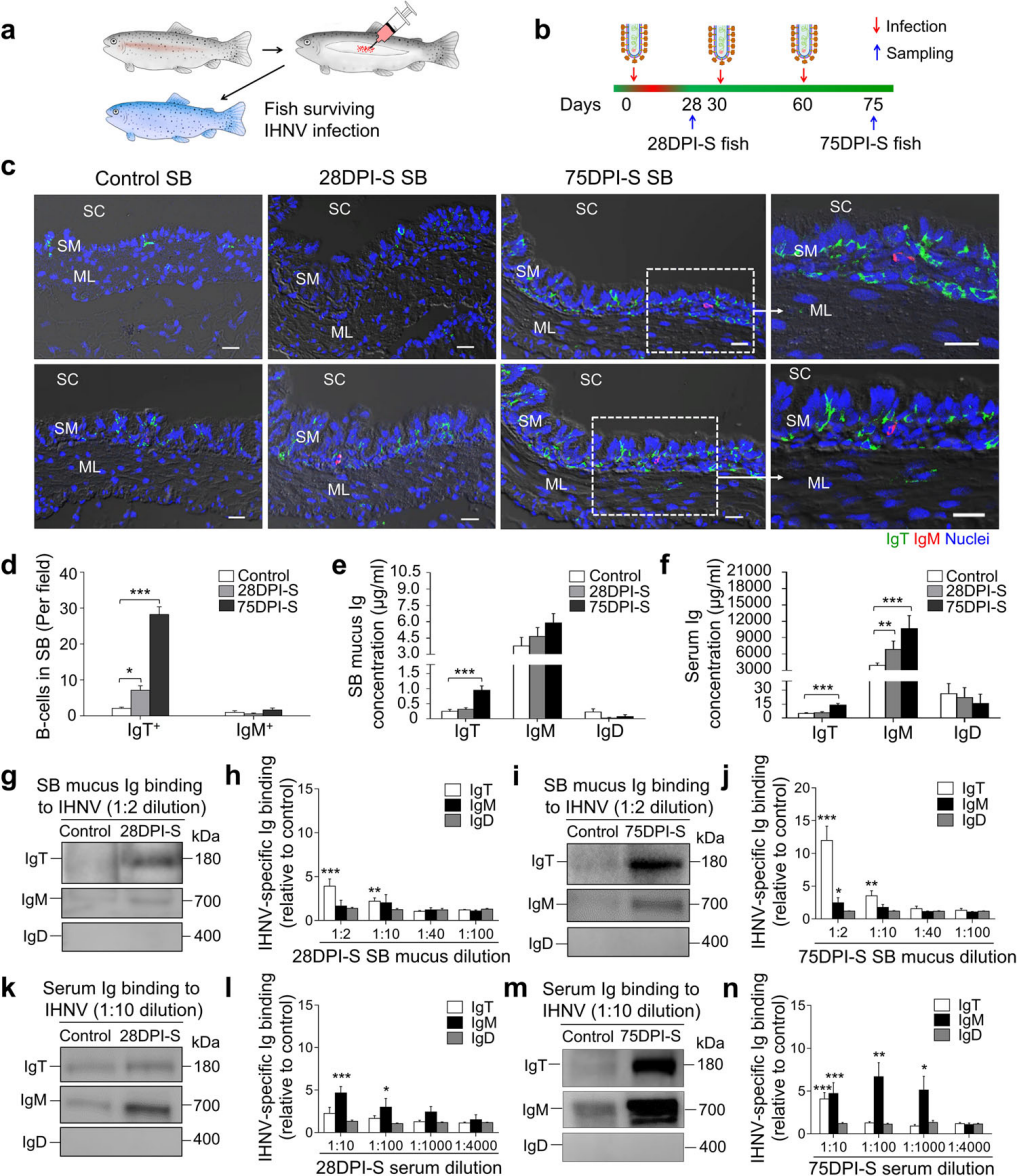
Increases in IgT^+^ B-cells and IHNV-specific sIgT responses in the SB mucosa of trout infected with IHNV. **a**, **b** Scheme of the infection strategy with IHNV via SB injection. Fish were injected with 25 μL of virus (1 × 10^5^ TCID_50_) and one group of surviving fish was sacrificed at 28 DPI (28DPI-S group) while another group was reinfected twice at 30 and 60 DPI with the same viral dose, and the resulting surviving fish were sacrificed at 75 days after the first infection (75DPI-S group). **c** Differential interference contrast (DIC) images of immunofluorescence staining on trout SB paraffin-sections from uninfected (control) fish, 28DPI-S and 75DPI-S fish, stained for IgT (green) and IgM (red) (Isotype-matched control antibody staining is shown in Supplementary Fig. S2c); nuclei (blue) were stained with DAPI (blue). SC SB cavity, SM SB mucosa, ML muscle layer. Scale bars, 20 μm. **d** The number of IgT^+^ and IgM^+^ B-cells in SB paraffin-sections of control, 28DPI-S and 75DPI-S fish counted from **c** (*n* = 9). **e**, **f** Concentration of IgT, IgM, and IgD protein in SB mucus (**e**) and serum (**f**) of control, 28DP-S, and 75DPI-S fish (*n* = 9–16). **g**, **i** Western blot analysis of IgT-, IgM-, and IgD-specific binding to IHNV in SB mucus (dilution 1/2) from 28DPI-S (**g**) and 75DPI-S fish (**i**). **h**, **j** IgT-, IgM- and IgD-specific binding to IHNV in dilutions of SB mucus from 28DPI-S (**h**) and 75DPI-S (**j**) fish evaluated by densitometric analysis of immunoblots and presented as relative values to those of control fish (*n* = 8–12). **k**, **m** Western blot analysis of IgT-, IgM-, and IgD-specific binding to IHNV in serum (dilution 1/10) from 28DPI-S (**k**) and 75DPI-S fish (**m**). **l**, **n** IgT-, IgM-, and IgD-specific binding to IHNV in dilutions of serum from 28DPI-S (**l**) and 75DPI-S (**n**) fish evaluated by densitometric analysis of immunoblots and presented as relative values to those of control fish (*n* = 8–12). Statistical differences were evaluated by one-way ANOVA with Bonferroni correction (**d**–**f**) and were performed by unpaired Student’s *t*-test (**h**, **j**, **l**, **n**). Data are representative of at least three independent experiments (mean ± SEM). *P < 0.05, **P < 0.01, and ***P < 0.001.

To evaluate adaptive viral-specific Ig responses, fish were challenged a second and third time with IHNV at 30 and 60 DPI respectively, using the same infection route and viral dose as the first infection (Fig. 4a, b). To assess the Ig and B-cell responses, samples were then collected at 28 (28 DPI surviving group, (28DPI-S)) and 75 (75 DPI surviving group, (75DPI-S)) days post-primary infection (Fig. 4b). As shown in Fig. 4c (isotype-matched control antibodies are shown in Supplementary Fig. S2c), very few IgT^+^ and IgM^+^ B-cells were present in the SB of control fish, demonstrated by immunofluorescence analysis. However, a moderate increase in IgT^+^ B-cells was detected in the 28DPI-S fish, and a notable accumulation of IgT^+^ B-cells was observed in the SB of the 75DPI-S group when compared to the control group (Fig. 4c, d). In contrast, the number of IgM^+^ B-cells did not change significantly in either of the surviving 28DPI-S or 75DPI-S group compared to control animals (Fig. 4c, d). In addition to these increments in IgT^+^ B-cells observed in surviving fish, the protein concentration of sIgT in the SB mucus of the 75DPI-S group increased by ~4-fold compared with that of control fish, whereas the protein levels of sIgM and sIgD remained largely unchanged (Fig. 4e). In serum, no significant change of IgT concentration was detected in 28DPI-S fish, while ~3-fold increase occurred in 75DPI-S fish, when compared with control fish. The concentration of serum IgM increased ~2- and 3-fold in 28DPI-S and 75DPI-S, respectively. In contrast, the IgD protein concentration did not change in either the SB mucus or serum of the same fish groups (Fig. 4e, f).

The aforementioned large increases in IgT^+^ B-cells and IgT protein levels in the SB tissue and mucus of surviving fish suggested the generation of virus-specific IgT immune responses. To verify this hypothesis, we measured the virus-specific titers of Igs in the SB mucus and serum via pull-down method in which purified IHNV was incubated with SB mucus or serum from control or surviving fish and then binding of sIg to the virus was analyzed by western blotting on pulled down ultracentrifuged viral particles. As shown in Fig. 4g–j, IHNV-specific IgT binding was detected in up to 1/10 dilution of SB mucus in both the 28- and 75DPI-S groups, while IHNV-specific IgM was only detected in the 1/2 mucus dilution in the 75DPI-S group. In serum, however, IgM was the dominant IHNV-specific Ig induced in surviving fish. Accordingly, significant increases in IHNV-specific IgM binding were observed in up to 1/100 and 1/1000 serum dilutions of 28DPI-S and 75DPI-S groups, respectively (Fig. 4k–n). In contrast, IHNV-specific IgT binding was detected only in up to 1/10 dilution of the 75DPI-S group (Fig. 4k–n). We could not detect any IHNV-specific IgD binding in the SB mucus or serum in either of the surviving fish groups (Fig. 4g–n). Exposure of fish with IHNV by immersion also led to a significant infection of the SB, and IgT-specific responses against IHNV (Supplementary Fig. S8), similar to the results when the virus was directly inoculated by injection into the SB (Fig. 4).

We next sought to determine whether after infection the pIgR would increase along with increased sIgT levels observed in the SB mucus. A significant number of pIgR-containing cells were associated with IgT staining in 75DPI-S fish (Supplementary Fig. S9a). Interestingly, the pIgR-protein levels increased markedly in the 75DPI-S group compared with those of control fish (Supplementary Fig. S9b, c), suggesting that the increase of sIgT in SB mucus of 75DPI-S group might require more pIgR in order to mediate its transport across the SB epithelium. Overall, these results indicate that sIgT is the dominant sIg isotype responding to the viral challenge in the SB and also the involvement of pIgR in the transport of sIgs across the SB epithelium.

### Local IgT^+^ B-cell proliferation and IgT production occurs in SB upon viral infection

**Fig. 5:**
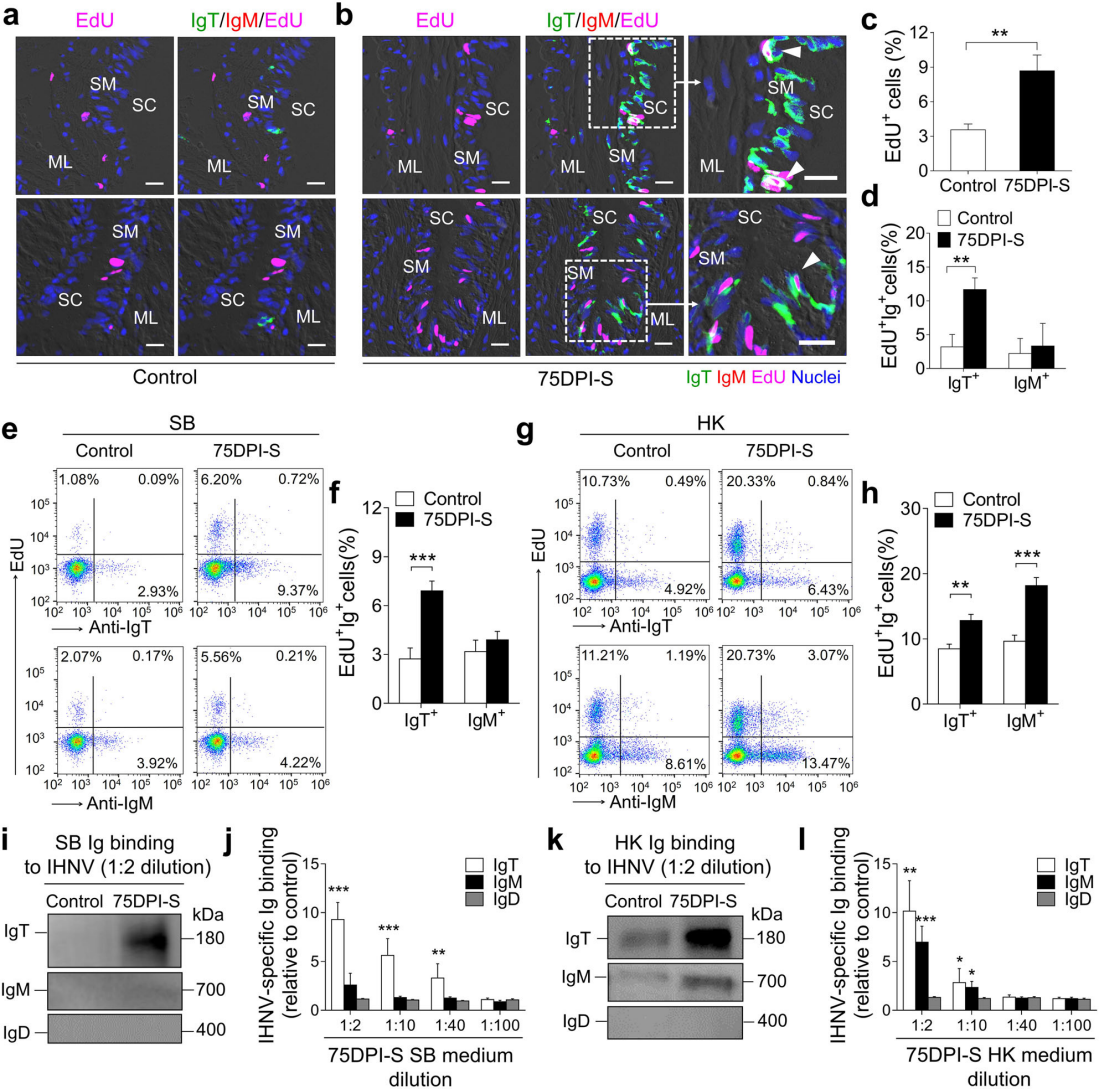
Proliferative responses of B-cells upon IHNV infection and local Ig-specific responses in tissues explants of 75DPI-S fish. **a**, **b** Immunofluorescence analysis of EdU incorporation by IgT^+^ or IgM^+^ B-cells in SB mucosa of control (**a**) and 75DPI-S (**b**) fish. SB paraffin-sections were stained for EdU (magenta), trout IgT (green), trout IgM (red), and nuclei (blue). White triangles within enlarged images of the areas outlined in **b** point to proliferative (double-stained for EdU and IgT) IgT^+^ B-cells (**b**, right). SC SB cavity, SM SB mucosa, ML muscle layer. Scale bars, 20 μm. **c**, **d** Percentage of EdU^+^ cells from total SB cells (**c**) and total IgT^+^ or IgM^+^ B-cells populations (**d**) in SB mucosa of control or 75DPI-S fish counted from **a** and **b** (*n* = 15). **e** Representative flow cytometry dot-plots showing proliferation of IgT^+^ and IgM^+^ B-cells in SB leukocytes of control and 75DPI-S fish. The percentage of lymphocytes representing proliferative B-cells (EdU^+^) is shown in each dot plot. **f** Percentage of EdU^+^ cells from the total SB IgT^+^ or IgM^+^ B-cell populations in control and 75DPI-S fish (*n* = 12). **g** Representative flow cytometry dot-plots showing proliferation of IgT^+^ and IgM^+^ B-cells in HK leukocytes of control and 75DPI-S fish. The percentage of lymphocytes representing proliferative B-cells (EdU^+^) is shown in each dot plot. **h** Percentage of EdU^+^ cells from the total HK IgT^+^ or IgM^+^ B-cell populations in control or 75DPI-S fish (*n* = 12). **i**–**l** Local IgT-, IgM- and IgD-specific responses in SB and HK explant supernatants from control and 75DPI-S fish. **i**, **k** Western blot analysis of IgT-, IgM-, and IgD-specific binding to IHNV in explant supernatants (dilution 1/2) of SB (**i**), and HK (**k**) from control and 75DPI-S fish. **j**, **l** IgT-, IgM-, and IgD-specific binding to IHNV in dilutions of explant supernatants of SB (**j**) and HK (**l**) from control and 75DPI-S fish, measured by densitometric analysis of immunoblots and presented as relative values to those of control fish (*n* = 8**–**10). Statistical differences were performed by unpaired Student’s *t*-test. Data in **c**, **d**, **f**, **h**, **j**, and **l** are representative of at least three independent experiments (mean ± SEM). *P < 0.05, **P < 0.01, and ***P < 0.001.

To investigate whether the increase of IgT^+^ B-cells observed in the SB mucosa of surviving fish was the result of local IgT^+^ B-cell proliferation, we assessed the in vivo proliferation of IgT^+^ and IgM^+^ B-cells stained with 5-Ethynyl-2’-deoxyuridine (EdU). Our immunofluorescence microscopy analysis indicated that there was a significant increase in overall cell proliferation in the SB mucosa of the 75DPI-S fish (8.74%) compared with that of controls (4.38%) (Fig. 5a–c). Importantly, the proliferation of IgT^+^ B-cells in the 75DPI-S fish showed a significant increase compared to that of the control group (Fig. 5a, b, d). However, no significant changes in IgM^+^ B-cell proliferation were observed in the SB mucosa between the 75DPI-S and control group (Fig. 5a, b, d). Similarly, flow cytometry analysis indicated that there was a significant increase in the number of EdU^+^IgT^+^ B-cells in the SB of the 75DPI-S fish (6.91%) compared to that of controls (2.73%), whereas no obvious changes in EdU^+^IgM^+^ B-cell numbers were observed between these groups (Fig. 5e, f). In contrast, the number of both EdU^+^IgT^+^ and EdU^+^IgM^+^ B-cells increased significantly in the HK of the surviving fish compared to that of controls, with the increases in EdU^+^IgM^+^ B-cell numbers being dominant (Fig. 5g, h). Additionally, we detected IHNV-specific Ig titers in tissue explant supernatants of SB and HK from control and 75DPI-S groups fish via pull-down assays. We found that the 75DPI-S group exhibited significantly higher IHNV-specific IgT titers (up to 1/40 dilution) but none for IgM and IgD in SB when compared to those of control fish (Fig. 5i, j). In contrast, significant IHNV-specific IgT and IgM titers (up to 1/10 dilution) were detected in the HK (Fig. 5k, l). Taken together, these results indicate that locally dominant IgT^+^ B-cells proliferation and IgT production occur in SB of fish surviving IHNV infection while in a systemic lymphoid organ, local IHNV-specific Ig responses were seen both for IgT and IgM isotypes.

### IHNV-specific IgT has a viral neutralizing capacity

**Fig. 6:**
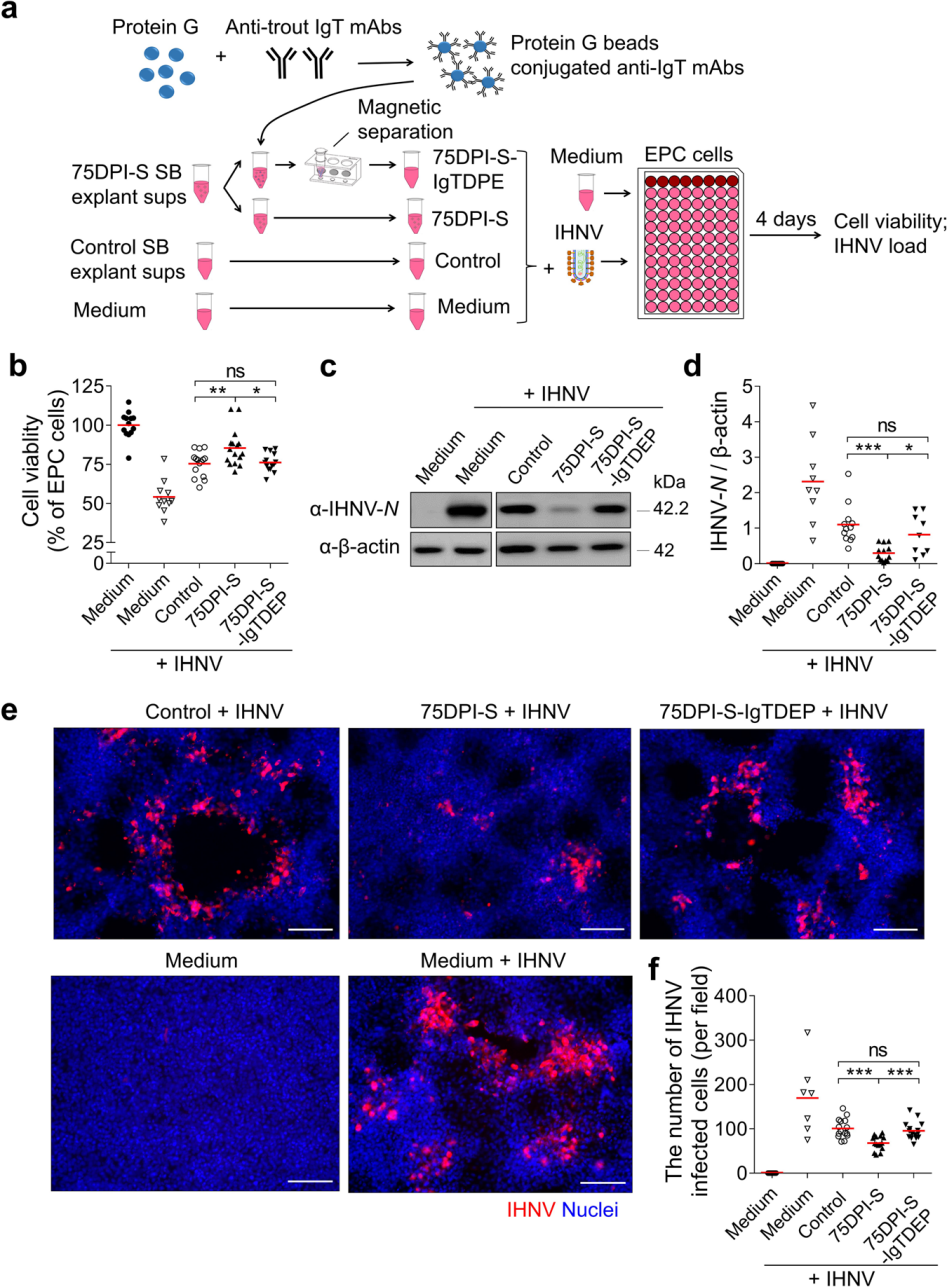
Viral neutralization exerted by IHNV-specific sIgT from SB of 75DPI-S fish. **a** Scheme of the experimental strategy. Magnetic protein G beads were incubated with anti-trout IgT mAbs to generate protein G beads coated with anti-IgT mAbs. SB explant supernatants (sups) from control and 75DPI-S fish were collected after 3 days incubation with MEM medium. IgT from 75DPI-S SB explant sups was depleted by incubating these sups with protein G beads coated with anti-IgT mAbs. Thereafter, IHNV was pre-incubated with medium alone or SB explant sups derived from control, 75DPI-S, or 75DPI-S-IgTDEP fish, and each of these IHNV-containing sups were added to EPC cells. Four days after addition of the different SB explant sups or medium alone to EPC cells, these five groups of EPC cell treatments (including: Medium (EPC cells), Medium + IHNV (IHNV-EPC cells), Control (control sups-IHNV-EPC cells), 75DPI-S (75DPI-S sups-IHNV-EPC cells), and 75DPI-S-IgTDPE (75DPI-S-IgTDPE sups-IHNV-EPC cells)) were analyzed for EPC cell viability and presence of IHNV-*N* protein. **b** The cell viability in the different groups were measured by the colorimetric alamarBlue assay and presented as relative values to those of EPC cells (*n* = 10–16). **c** The IHNV-*N* protein expression in EPC cells was detected by western blot using an anti-IHNV-*N* mAb. **d** Relative expression of IHNV-*N* protein in EPC cells was evaluated by densitometric analysis of immunoblots and presented as relative values to those of control fish (*n* = 9–13). **e** The IHNV-*N* protein in EPC cells was detected by immunofluorescence using the anti-IHNV-*N* mAb (Isotype-matched control antibody staining is shown in Supplementary Fig. S2c). Scale bars, 100 μm. **f** Numbers of virally infected cells were calculated from **e** (*n* = 7–15). Each symbol (**b**, **d**, **f**) represents an individual fish; small horizontal red lines (**b**, **d**, **f**) indicate the means. Statistical differences were evaluated by one-way ANOVA with Bonferroni correction. Data in **b**, **d**, and **f** are representative of at least three independent experiments (mean ± SEM). *P < 0.05, **P < 0.01, and ***P < 0.001.

Our findings showing that IHNV infection resulted in a significant IHNV-specific IgT titer in the SB mucus (Fig. 4) and that fish re-exposed to IHNV several times were protected against the virus (Supplementary Fig. S6) lead to the hypothesis that IHNV-specific IgT could have a neutralizing viral capacity in SB. To evaluate this hypothesis, IHNV was incubated for 1 h with several solutions, including medium, and SB tissue explant supernatants (sups) from control and 75DPI-S fish. Importantly, the role of IgT was evaluated by prior depletion of IgT^+^ B-cells from the 75DPI-S sups, which led to 75DPI-S sups devoid of IgT (75DPI-S-IgTDEP sups) (Fig. 6a). After incubation, these four different IHNV-containing sups, as well as cell culture medium alone (as control), were incubated with EPC cells, and thereafter, EPC cell viability and IHNV load were assessed in all EPC cell cultures (Fig. 6a). We found that EPC cell viability was higher when using the sups from 75DPI-S compared with that of control fish (Fig. 6b), indicating a protective effect of the sups from these survivor fish. Conversely, the viability of EPC cells treated with 75DPI-S-IgTDEP sups was similar to that of control non-immune fish, indicating that the protective effect of the 75DPI-S sups was due to IHNV-specific IgT contained in these sups (Fig. 6b). In line with these results, we found that the expression of IHNV*-N* protein levels (Fig. 6c, d), as well as the number of virally infected EPC cells (Fig. 6e, f; IHNV-*N* mAb isotype control antibodies are shown in Supplementary Fig. S2d), decreased markedly in EPC cells treated with the 75DPI-S SB sups while these levels increased significantly to levels observed for SB sups of control fish when IgT^+^ B-cells were depleted in the 75DPI-S SB sups (i.e., 75DPI-S-IgTDEP sups). Taken together, these results strongly indicated a key role of SB sIgT in IHNV neutralization.

### The SB of IgT^+^ B-cell-depleted fish had an increased susceptibility to IHNV infection

We have described above that trout infected with IHNV generates strong local viral-specific sIgT responses in the SB mucosa (Fig. 5i, j) and that IHNV-specific IgT has neutralizing antiviral activity (Fig. 6b–f). These data suggested a protective role of IgT against IHNV. To determine whether sIgT plays a protective role in the SB mucosa against IHNV, an IgT^+^ B-cell depletion model previously established by us^27^ was utilized to evaluate IHNV loads in the SB of IgT^+^ B-cell-depleted and non-depleted fish. We first evaluated the degree and duration of IgT^+^ B-cells and IgT depletion in SB tissue and SB mucus respectively of IgT^+^ B-cell-depleted and control fish (Supplementary Fig. S10). Flow cytometry analysis indicated that IgT^+^ B-cells were significantly depleted in the SB tissue from 7 days post-depletion treatment, and that this depletion persisted for several weeks to a high level (54%–86% of depleted IgT^+^ B-cells) when compared with that of control fish (Supplementary Fig. S10a). In contrast, no differences in IgM^+^ B-cell numbers were observed in the same fish (Supplementary Fig. S10b). Moreover, significant reductions (87%–89%) in IgT protein levels were observed in SB mucus from day 7 post-depletion treatment (Supplementary Fig. S10c). Further, unlike the control group, low sIgT levels in the depleted fish persisted for several weeks while recovering to those of control levels by day 42 (Supplementary Fig. S10c). In contrast, the IgM and IgD levels remained unaltered after post-depletion treatment (Supplementary Fig. S10d, e).

**Fig. 7:**
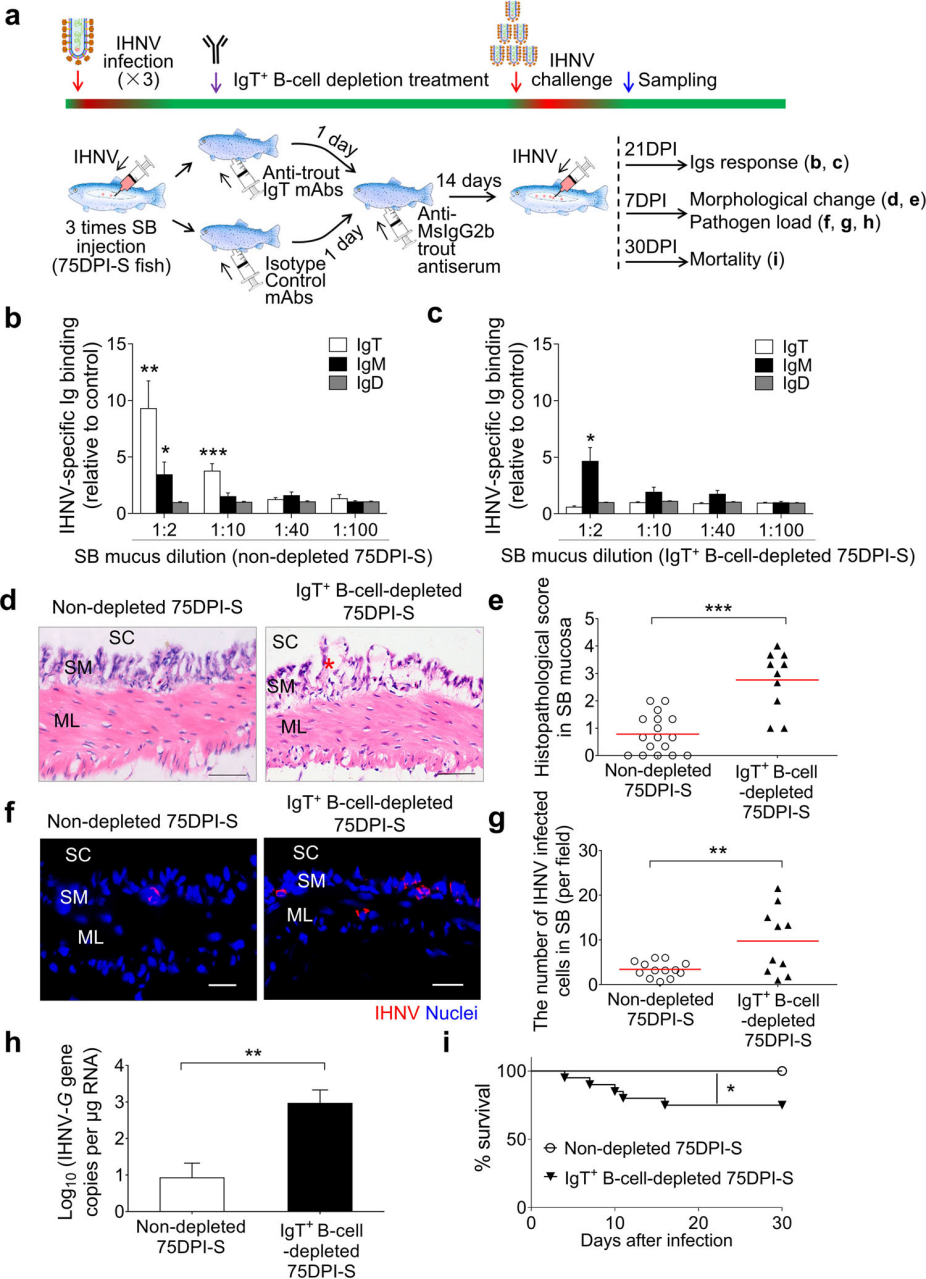
IgT^+^ B-cell depletion in fish significantly increases SB viral load and fish mortalities upon IHNV infection. **a** Scheme of the experimental strategy. Briefly, immune fish (fish that survived monthly IHNV infections via SB injection, during a 3-month period (75DPI-S fish)) were injected with either isotype (mouse IgG2b) control Abs (non-depleted 75DPI-S fish) or anti-IgT mAb (IgT^+^ B-cell-depleted 75DPI-S fish). One day later, fish were injected with anti-mouse IgG2b trout antiserum. At 14 days after trout antiserum injection, the non-depleted 75DPI-S and IgT^+^ B-cell-depleted 75DPI-S groups were infected by intra-SB injection with IHNV (25 μL, 1 × 10^7^ TCID_50_), and at 21, 7, and 30 DPI, the two fish groups were analyzed for Ig responses, morphological SB changes and SB viral load, and mortalities, respectively. **b**, **c** IgT-, IgM-, and IgD-specific binding to IHNV in dilutions of SB mucus from non-depleted (**b**) and IgT^+^ B-cell-depleted (**c**) 75DPI-S fish at 21 DPI. Results are presented as relative values to those of the SB mucus from uninfected naive fish (*n* = 8–12). **d** H&E staining of trout SB from non-depleted and IgT^+^ B-cell-depleted 75DPI-S fish at 7 DPI. Red asterisk indicates the damage in SM. Scale bars, 50 μm. SC SB cavity, SM SB mucosa, ML muscle layer. **e** Pathology score of SB mucosa in non-depleted and IgT^+^ B-cell-depleted 75DPI-S fish at 7 DPI. **f** IHNV protein was detected by anti-IHNV-*N* protein mAb (red) in SB mucosa from non-depleted (left) and IgT^+^ B-cell-depleted (right) 75DPI-S fish at 7 DPI. Nuclei were stained with DAPI (blue). SC SB cavity, SM SB mucosa, ML muscle layer. Scale bars, 20 μm. **g** The number of virally stained cells in SB mucosa of non-depleted and IgT^+^ B-cell-depleted 75DPI-S fish upon challenge with IHNV counted from **f**. **h** Upon challenge, IHNV-*G* gene copies (Log10) was quantified using qPCR in SB from non-depleted and IgT^+^ B-cell-depleted 75DPI-S fish at 7 DPI (*n* = 10). **i** Cumulative survival of non-depleted and IgT^+^ B-cell-depleted 75DPI-S fish infected with IHNV. Each symbol (**e**, **g**) represents an individual fish; small horizontal red lines (**e**, **g**) indicate the means. Statistical differences were evaluated by unpaired Student’s *t*-test (**b**, **c**, **e**, **g**, **h**) and log-rank (Mantel-Cox) test (**i**). Data are representative of at least three independent experiments (mean ± SEM). *P < 0.05, **P < 0.01, and ***P < 0.001.

After demonstrating that the IgT^+^ B-cell depletion strategy was instrumental to deplete IgT in the SB, we next asked whether the SB from surviving fish would become more susceptible to IHNV infection upon IgT^+^ B-cell depletion treatment. As previously shown, the SB mucus from 75DPI-S fish exhibited a significant IgT-dependent neutralization activity against the virus, and thus, we hypothesized that IgT^+^ B-cell depletion would make the SB from 75DPI-S fish highly susceptible to the virus. To test this hypothesis, 75DPI-S fish were divided into two groups. One group (IgT^+^ B-cell -depleted 75DPI-S group) underwent IgT^+^ B-cell depletion treatment (as shown in Fig. 7a), whereas the other (non-depleted 75DPI-S group) was treated with isotype control Abs. At 14 days post-treatment, both depleted and non-depleted groups were challenged with a high dose of IHNV (25 μL, 1 × 10^7^ TCID_50_) after which viral load, SB-tissue morphological changes, Igs responses, and fish mortalities were recorded at 7, 21, and 30 days post-challenge respectively (Fig. 7a). As expected, the non-depleted 75DPI-S group contained significant levels of IHNV-specific sIgT titers in the SB mucus (Fig. 7b), whereas the IgT^+^ B-cell-depleted 75DPI-S group did not (Fig. 7c). In contrast, IgT^+^ B-cell depletion did not have an effect on the virus-specific IgM titers present in the mucus (Fig. 7b, c). More importantly, the IgT^+^ B-cell-depleted 75DPI-S group had significant mucosa damage in the SB and the viral load in the same organ increased markedly when compared to the non-depleted group (Fig. 7d–g). These results were consistent with the higher expression of the IHNV-*G* gene in the SB of the IgT^+^ B-cell-depleted fish (Fig. 7h). Additionally, upon challenge with IHNV, the IgT^+^ B-cell-depleted 75DPI-S group had a significantly higher cumulative mortality compared with that of non-depleted fish (Fig. 7i). Taken together, our results confirmed the critical role of sIgT in protecting the SB mucosa against IHNV.

## Discussion

The AOs of vertebrates have adapted to different living environments and have evolved unique functions to ensure the survival of the species. More specifically, in teleosts, SB evolved buoyancy control functions, whereas basal lobe-finned fishes (like lungfish) and terrestrial organisms developed lungs that enabled them to breathe air. When performing their functions, both specialized AOs were subjected to comparable selective forces from airborne or aquatic pathogens and commensals on their mucosal surfaces, and thus, they may have been evolved similar defense strategies. However, immunity in AOs has only been studied in tetrapod lungs, while little is known about the roles of teleost AOs (i.e., SB) in host defense. To gain insights into the evolution of AOs immune responses, here we evaluated whether the AOs of an aquatic species (i.e., teleost SB) could fulfill immune roles.

Our data show that the trout SB mucosa contains a well-defined diffuse MALT characterized by an epithelium layer, which contains most of the canonical features of other teleost MALTs^37,39^. Accordingly, the SB mucosa lacks organized lymphoid structures and instead exhibited scattered lymphoid cells, where IgT^+^ B-cells were the predominant B-cell type. In addition, we observed that pIgR-positive cells exist in the epithelial layer of the SB mucosa and that trout tSC was associated with sIgT in the SB mucus. Similarly, in mammalian lungs, gut, uterus, and nasal mucosa, pIgR is required for the transport of sIgA into luminal areas, thus playing a critical role in the immune response against pathogens^32–35^. Accordingly, our results show that SB mucosa harbors an sIg transport mechanism that is remarkably similar to that of other teleost MALTs and mammalian type-I mucosal surfaces. A feature of the teleost MALT common to that of other vertebrate MALTs is the presence of a diverse and dense microbiota and coating of commensals by mucosal Ig^37^. To prevent the microbiota that populates the mucus from gaining access into the mucosal epithelium, sIgA acts through an immune exclusion mechanism in mucosal surfaces by coating different commensal bacteria^40,41^. Here we determined that sIgT is the main Ig class that coats the SB microbiota, while a smaller portion of the bacteria was coated by sIgM and sIgD as has been shown also for other trout MALTs^22,42^. Interestingly, we found that the SB represented the trout MALT with the lowest concentration of bacteria, a feature that resembles that of the lung. Accordingly, while in the past it was generally accepted that healthy mammalian lungs were sterile (i.e., devoid of microbiota), a growing body of research using novel microbial identification techniques has demonstrated that the lungs harbor diverse microbial communities^43^, although their density and diversity is significantly lower than that of the gut, or the upper respiratory tract^44^. To the best of our knowledge, our findings provide the first demonstration that sIgs coat the microbiota of a vertebrate AO surface.

In order to evaluate the capacity of the SB to behave as an immune-responsive tissue, we established an infection model in which IHNV is quickly and specifically delivered into the trout SB via an intra-SB cavity injection. A similar strategy has been used to inoculate the SB of larval zebrafish with a number of pathogens, including *Candida albicans*^45^, *Mucor circinelloides*^29^, *Candida albicans*/*Pseudomonas aeruginosa* coinfection^30^, influenza A^46^, and SARS-CoV-2 virus^28^. Here, our results show that upon delivery of the virus into the SB cavity, a significant increase in IHNV SB load was observed in diseased fish as early as 4 days, while other distant tissues (such as skin) had very few viral copies. Our results demonstrate that IHNV infection can infect the SB and cause interstitial tissue damage in rainbow trout. More importantly, viral progeny could be reisolated from diseased SB, supporting the feasibility and validity of the infection model. Previous studies in zebrafish have suggested a role of SB in innate immunity^29,47^. Accordingly, it has been reported that SB inoculated with fungal pathogens or lipopolysaccharide via intra-SB injection had increased numbers of macrophages and neutrophils as well as increased mRNA expression of IL-6 and TNF-α^29,48^. Here we confirm and expand the innate immune capabilities of the SB in innate immunity, and describe a previously unrecognized capacity of the SB to express and upregulate antiviral genes in response to viral infection. More specifically, we show that the trout SB mucosa exhibited an upregulation of antiviral genes (RIG-I, LGP2, Mx1, IRF7, and MDA5) that are known to be essential for the production of effective antiviral responses in the mammalian type-I mucosal surfaces, such as lung, gut, and nose, particularly at early stages of infection (i.e., 4 and 7 DPI)^49–51^. Moreover, IHNV challenge resulted in significant upregulation in the expression of chemokine genes, such as CXCL9 and CXCL11, which are involved in the recruitment of lymphocytes to inflammatory sites in mammals^52^. The transcriptomic analysis further demonstrated that a very large number of innate and adaptive immune genes were expressed and upregulated upon viral infection, thus indicating an important role for SB in immune responses. More critically, we demonstrate that in addition to B cells, IHNV infection elicited significant upregulation in the mRNA expression of T helper cell- and macrophage-specific surface makers, and thus, these results in combination with the data provided by transcriptome strongly suggest that the fish SB is a real MALT. More importantly, we also demonstrate the induction of adaptive mucosal immune responses in the SB MALT. More specifically, we found that a large accumulation of IgT^+^ but not IgM^+^ B-cells occurred in the SB epithelium of survivor fish, which correlated with increases in IgT concentration but not IgM or IgD in the SB mucus of the same individuals. Similarly, numerous IgA-secreting plasma cells, in addition to increases in sIgA concentration, occur in mammalian MALTs, such as GALT, NALT, and BALT after rotavirus, reovirus, and pulmonary virus infection, respectively^14,53,54^. Confirming the induction of IHNV-specific mucosal immune responses, virus-specific IgT, and to a lesser degree, IgM titers were detected in the SB mucus, whereas high and dominant virus-specific IgM responses were only seen in the serum. These findings are similar to results obtained in several MALTs from fish infected with parasitic or bacterial pathogens^22^, suggesting that IgT and IgM responses are mainly confined to mucosal and systemic tissues, respectively. Importantly, our study is the first to demonstrate that a viral pathogen elicits dominant IgT responses in fish mucosal tissue. Interestingly, a recent study has shown that sIgA is the predominant isotype in the lungs of patients with COVID-19, especially in the early immune response^19^. Moreover, predominant virus-specific IgA responses are induced in the intestine and nasal mucosa after infection with rotavirus and influenza virus, respectively^55,56^. Therefore, our results indicate that both modern and primitive mucosal sIgs (i.e., IgA and IgT) are induced in response to viral pathogens in lungs and SB, thus pointing to an evolutionary-conserved role of these sIgs in the defense of AOs against viruses.

In mammalian lungs, B-cells first proliferate at the inductive site (e.g., lung-mediastinal lymph nodes and induced bronchus-associated lymphoid tissue), after which they migrate to the effector site, where sIgs are secreted by local plasma cells into the lungs^57^. Given the lack of lymph nodes containing germinal centers (GCs) in teleost fish, it is unclear whether the accumulation of IgT^+^ B-cells observed in the SB mucosa is the result of local B-cell proliferation or migration from other lymphoid organs. Here, we found significant proliferative IgT^+^ B-cell responses in the SB mucosa of trout after infection with IHNV, similar to what we have previously been reported in other fish mucosa infected with different pathogens^25,26^. Moreover, we detected strong IgM^+^ and moderate IgT^+^ B-cell proliferative responses in the HK of the same fish. Our results suggest that the accumulation of IgT^+^ B-cells in the SB mucosa is the result of their local proliferation, while a relatively small fraction may derive from the HK or other lymphoid organs, although this hypothesis remains to be fully demonstrated. Additionally, we detected virus-specific IgT titers in SB tissue explant cultures. Although these data further suggest that IgT-specific responses are induced locally in the SB mucosa, we cannot rule out the possibility that some activated IgT^+^ B-cells in the SB mucosa migrated from systemic lymphoid tissues and/or other mucosal tissues through the vascular system instead of proliferating locally, after which they differentiated into plasma cells in the SB to produce virus-specific IgT. Therefore, additional studies are required to address the detailed mechanisms underlying the local production of IgT-specific responses in the SB mucosa. It is also worth noting that influenza hemagglutinin-specific IgA is produced locally in the mice lung tissue^58–60^.

We found that survivor fish re-exposed to the virus survived the infection, suggesting the generation of protective immune responses in these fish. This led to the hypothesis that the virus-specific IgT responses had an antiviral neutralizing capacity, which was supported by the results that IgT^+^ B-cell-depleted SB mucus incubated with EPC cells cultures significantly lost its capacity to protect EPC cells from viral infection. Thus these data support a previously unrecognized role for sIgT in viral neutralization. Similarly, IgA-mediated viral neutralization has been demonstrated for several viruses affecting the lungs and other mucosal surfaces in humans, e.g., sIgA-mediated neutralization of SARS-CoV-2 in the lungs^19^. Moreover, locally produced IgA in the human upper respiratory mucosa plays a pivotal role in virus neutralization and protection against influenza virus infection^61,62^.

To further determine the role of sIgT in IHNV resistance, we selectively depleted IgT^+^ B-cells in rainbow trout, using a novel strategy recently described by us^27^. Critically, we found that upon depletion of IgT^+^ B-cells from fish immune to the virus (i.e., survivor fish previously exposed to IHNV), their SB mucosa became highly susceptible to IHNV infection, which led to severe SB epithelium damage and high viral loads in the SB tissue. In contrast, the non-depleted survivor fish had markedly lower viral loads and did not present any pathological damage in their SB tissue. These data clearly indicated that IgT plays a key role in host defense against viral infections in the SB, thus supporting our data on the neutralizing capacity of virus-specific IgT. Moreover, IgT^+^ B-cell-depleted fish exhibited significant mortality rates one month after reinfection compared with the non-depleted group, thus indicating that IgT is critical for immune defense against IHNV. Previous studies in mammals have shown similar results with regards to the role of sIgA in antiviral defense. For example, IgA^*−/−*^ mice failed to develop protective immunity against multiple repeated exposures to rotavirus infection in gut, whereas wild-type mice were completely protected against reinfection^63^. In addition, respiratory infections in patients with sIgA deficiency are more recurrent in their lungs^64^. Thus, our findings provide the first demonstration that a non-mammalian mucosal sIg plays a key role against a viral pathogen.

Here we also show that similar to other teleost mucosal surfaces^22,37^, the SB contains a significant microbiota population that is prevalently coated by sIgT, and to a much lesser degree by IgM and IgD. Interestingly, the amount of microbiota in the SB was the lowest of all fish MALTs, a situation that resembles that of the lungs from tetrapods which contain a very small amount of microbiota when compared to other mucosal surfaces^44,65,66^. Future studies are warranted to evaluate the role of sIgT in SB microbiota homeostasis.

In conclusion, in addition to its well-known role in buoyancy control, our results reveal a previously unrecognized function of teleost SB in adaptive mucosal immunity. Importantly, we showed pathogen-specific IgT production within the SB of fish and demonstrated the capacity of specific IgT in viral neutralization, thus firstly discovered the critical role of sIgT in viral neutralization in teleost fish. Moreover, the role of SB in mucosal immunity was further supported by the coating of SB microbiota particularly by sIgT, thus suggesting a role for SB sIgT in microbiota homeostasis. Overall, our results provide insight into the evolution of immune responses to pathogens and microbiota in an ancient AO, by providing evidence that vertebrate AOs and specialized mucosal Igs are part of an ancient partnership that predates the emergence of tetrapods.

## Methods

### Ethics statement

All experimental protocols were performed in accordance with the guidelines and regulations established in the Guide for the Care and Use of Laboratory Animals of the Ministry of Science and Technology of China. The protocols were approved by the Scientific Committee of Huazhong Agricultural University (permit number HZAUFI-2017-001). All animal procedures involving experiments performed at the University of Pennsylvania were approved by the Institutional Animal Care and Use Committees of the University of Pennsylvania. All efforts were made to minimize the suffering of the experimental fish.

### Fish maintenance

All experimental rainbow trout were obtained from a fish farm in Shiyan (Hubei, China) and Troutlodge (Summer, WA), and maintained and acclimatized in an aerated recirculating aquaculture system at 16 °C with an internal biofilter. Fish were fed daily with dry pellets at 1% biomass/day, and fasted two days prior to injection and sampling. Zebrafish (*Danio rerio*) samples were kindly provided by Dr. Chunsheng Liu (Huazhong Agricultural University, Wuhan, China). Atlantic salmon (*Salmo salar*) and Silver arowana (*Osteoglossum bicirrhosum*) were purchased from Huamucheng Market (Wuhan, China).

### Infection of fish with IHNV and sample collection

The infection of trout SB with IHNV was performed following a strategy similar to that reported for the intra-SB injection delivery of pathogens to juvenile zebrafish SB^28,29,45,46^. Briefly, IHNV was intra-SB injected into the SB lumen of trout (~10 g) by inserting the needle between the lateral line and the base of the anal fin at a 30- to 45-degree angle for a depth of 0.5–1 cm as shown in Supplementary Fig. S4a. Two types of challenges with IHNV were performed to generate two groups of fish, the first group representing fish exposed only once to the virus, while in the second group fish were exposed several times to IHNV. In the first group, 25 μL of IHNV (1 × 10^5^ TCID_50_) was intra-SB injected into the SB lumen. At 1, 4, 7, 14, 21, and 28 DPI, fish from this first group were euthanized with an overdose of tricaine methanesulfonate (MS-222, Sigma), and tissues including HK, spleen, skin, gut, and SB were collected for the detection of immune gene expression, viral load, and pathological changes. For the second type of challenge, the animals surviving from the primary infection were challenged a second and a third time with the same dose of IHNV at 30 and 60 DPI respectively. These fish were let to recover from infection (survivor fish). Fish surviving 28 DPI (28DPI-S fish) from the first group and 75 DPI from the first injection of the second group (75DPI-S fish) were sacrificed, and samples including SB, HK, and serum were collected from both groups. Mock-infected (uninfected) fish were intra-SB injected with the same amount of culture medium from uninfected EPC cells. SB mucus was collected with a similar method as that we previously described^26^. Briefly, the SB organ was opened up longitudinally and incubated for 12 h at 4 °C, with occasional shaking in a protease inhibitor buffer (1× protease inhibitor cocktail (Roche), 1 mM phenylmethylsulfonyl fluoride (Sigma) in PBS, pH 7.2) at a ratio of 0.1 g of SB tissue per mL of the buffer. The suspension was collected, vigorously vortexed, and then centrifuged at 400 × *g* for 10 min at 4 °C to remove the SB debris and cells, and the resulting supernatant comprised the SB mucus containing bacteria. To separate SB bacteria from mucus, the cell-free supernatant was thereafter centrifuged at 10,000 × *g* for 10 min at 4 °C. The resulting supernatant (SB mucus) was harvested, filtered with a 0.45 μm syringe filter (Millipore) and stored at 4 °C prior to use the same day or −80 °C until further use, whereas the pellet (SB bacteria) was washed three times with PBS (pH 7.2) and resuspended in the same buffer for further analysis.

### Binding of trout Igs to IHNV

To further assess whether the infected or survivor fish had generated IHNV-specific Igs, we measured the capacity of IHNV-specific IgT, IgM, and IgD in the SB mucus, serum or tissue (SB and HK) explant supernatants to bind to IHNV by a pull-down assay strategy similar to that described by us^26^. Briefly, the aforementioned fluid samples from control and survivor fish were first centrifuged at 100,000× *g* for 10 min at 4 °C to remove tiny tissue particles and debris. To purify the IHNV, the aforementioned IHNV suspension was thereafter ultracentrifuged over a 20% sterile sucrose layer at 100,000× *g* for 2 h at 4 °C by using the Optima L-100XP (Beckman Coulter) ultracentrifuge. The resulting pellet (purified IHNV) was resuspended in PBS and its total protein concentration was determined by the Bradford protein assay according to the manufacturer’s instructions (Quick Start™ Bradford Protein Assay kit, Bio-Rad). For the pull-down assay, 20 μL of purified IHNV (~30 μg/mL) was pre-incubated with 1% BSA (wt/vol) in PBS (pH 7.2; PBS-BSA) at 4 °C for 2 h, and thereafter incubated with serially diluted SB mucus, serum or tissue explant supernatants in PBS-BSA in a 300 μL volume reaction. After 12 h of incubation with occasional shaking at 4 °C, the IHNV were separated by ultracentrifugation as described above and bound proteins were eluted with 2× Laemmli Sample Buffer (Bio-Rad), and boiled for 5 min at 95 °C. The eluted material was resolved on 4%–15% SDS-PAGE Ready Gel under non-reducing conditions, and the presence of IgT, IgM, or IgD was detected by western blotting as described in Supplemental Methods.

### Trout SB IgT neutralization to IHNV

To evaluate the viral neutralizing capacity exerted by IHNV-specific IgT in SB from 75DPI-S fish, we developed an in vitro IgT^+^ B-cell depletion strategy to remove sIgT from SB explant supernatant (75DPI-S-IgTDEP sups). To this end, we generated anti-IgT-conjugated protein G beads by conjugating ~4 μg of mouse anti-trout IgT mAb (clone 41.8; IgG2b)^23^ to 200 µL protein G magnetic beads (Biolinkedin). To absorb sIgT, the anti-IgT-conjugated protein G beads were applied into 100 µL 75DPI-S sups and incubated with shaking for 2 h at 18 °C. Thereafter, the beads were separated from the supernatant by MagnaBind magnets (Biolinkedin), and the resulting supernatant was used as 75DPI-S-IgTDEP sups. The removal of sIgT in 75DPI-S-IgTDEP sups was confirmed by western blot analysis. To ensure sterility for the virus capture and neutralization assays, the control, 75DPI-S and 75DPI-S-IgTDEP sups were passed through a 0.22 μm Millex GV filter (Millipore). For neutralization assay, 100 µL of IHNV (2 × 10^4^ TCID_50_) in MEM was incubated with 100 µL of control (Control + IHNV), 75DPI-S (75DPI-S + IHNV), and 75DPI-S-IgTDEP (75DPI-S-IgTDEP + IHNV) sups at 18 °C for 1 h. The mixtures were then applied to infect EPC cells in 96-well plates (2 × 10^4^ cells per well) and incubated at 18 °C for 2 h. MEM with IHNV (Medium + IHNV) or without (Medium) was added to EPC cells as controls. Thereafter, EPC cells were washed twice with PBS, and then cultured at 18 °C in 5% FBS/MEM. At 4 days post-application, the infected EPC cells were collected to detect the viability of infected EPC cells and the presence of IHNV protein *N* as described in Supplemental Methods.

### In vivo depletion of IgT^+^ B-cells and IgT in SB tissue and mucus

The IgT^+^ B-cell-depleted fish was generated following a similar strategy to that previously described by us^27^. Briefly, fish (~10 g) were intraperitoneally injected with 75 μg of anti-trout IgT mAbs or mouse IgG2b as isotype control antibody (Biolegend). After 24 h, fish were further injected with 25 μL of trout anti-mouse IgG2b antisera (titer ≥ 1/128,000) to improve the depletion efficiency^27^. To evaluate the degree and duration of IgT^+^ B-cell depletion, SB tissues were obtained from these fish at 7, 14, 21, 28, 42, and 63 days after Ab injection. IgT^+^ and IgM^+^ B-cell populations from SB were analyzed by flow cytometry, and the concentration of Igs in the SB mucus was measured by western blot analysis as described in Supplemental Methods. To evaluate the susceptibility to IHNV of fish depleted of IgT^+^ B-cells, one group of 75DPI-S fish was treated for IgT^+^ B-cell depletion (IgT^+^ B-cell-depleted fish) as described above, whereas the other group was treated with isotype control Abs (non-depleted fish). Fourteen days after the depletion treatment, non-depleted and IgT^+^ B-cell-depleted groups were challenged with 25 μL of IHNV (1 × 10^7^ TCID_50_) by intra-SB injection. At 7 days after infection, SB tissues were obtained from both groups of fish. The IHNV loads in SB tissues were examined via qPCR and immunofluorescence analyses. Morphology changes in SB tissues were measured by H & E staining. On day 21 post-IHNV challenge, serum and SB mucus were collected to measure the IHNV-specific Ig responses by pull-down assays as described above.

### Statistical analysis

An unpaired Student’s *t*-test, one-way analysis of variance with Bonferroni correction, and log-rank (Mantel-Cox) test (Prism version 6.0; GraphPad) were used for the analysis of differences between groups. *P*-values of 0.05 or less were considered statistically significant.

## Supplementary information

Supplementary Information

Supplementary Video S1
