# Supplementary material for: Teleost swim bladder, an ancient air-filled organ that elicits mucosal immune responses
Source: Cell Discov. 2022 Apr 5;8:31. doi: 10.1038/s41421-022-00393-3 (PMC8979957; doi:10.1038/s41421-022-00393-3)
Supplement: Supplementary file 1 — Supplementary Information [file 41421_2022_393_MOESM1_ESM.pdf]

Supplementary Information for

Teleost swim bladder, an ancient air-filled organ that elicits mucosal immune responses

Yongyao Yu[1], Zhenyu Huang[1], Weiguang Kong[1], Fen Dong[1], Xiaoting Zhang, Xue Zhai, Gaofeng Cheng, Mengting Zhan, Jiafeng Cao, Liguo Ding, Guangkun Han, Fumio Takizawa, Yang Ding, J. Oriol Sunyer[*], Zhen Xu[*]

[1]These authors contributed equally

[*]Correspondence author: Zhen Xu (zhenxu@ihb.ac.cn), J. Oriol Sunyer (sunyer@vet.upenn.edu)

This PDF file includes:

Supplementary Methods

Supplementary References

Supplementary Figures S1-S11

Supplementary Table S1

Legend for Supplementary Video S1

Supplementary Methods

Cell culture and virus titration. The IHNV was propagated and titrated in epithelioma papulosum cyprini (EPC) cells as previously reported[1]. Briefly, EPC cells were maintained in Minimum Essential Medium with Earl's salts (MEM, Gibco) supplemented with 10% fetal bovine serum (FBS, Gibco), 100 µg/mL streptomycin, and 100 U/mL penicillin (Gibco) with 5% CO_2 at 28 °C. The cell density was monitored until it reached 70-90% confluence in a T-75 flask (Corning) prior to viral infection. 10 µL of IHNV (1×10^7 $TCID_{50}$) was inoculated to these cells in 12 mL culture medium and incubated at 18 °C for 2 hours. Thereafter, the media was replaced with 12 mL of 5% FBS/MEM. When extensive cytopathic effects (CPEs) were observed, the infected cells and medium were submitted to three cycles of freezing and thawing, followed by a gradient centrifugation (400 *g* for 10 min, 3,000 *g* for 10 min, and 10,000 *g* for 10 min) at 4 °C. The supernatant containing IHNV was filtered with a 0.22 µm PES membrane filtration (Millipore) to remove cell debris and bacteria, and titrated by the Median Tissue Culture Infectious Dose ($TCID_{50}$) assay on EPC cells following the methodology reported by Reed and Muench[2]. Briefly, IHNV suspension was 10-fold serially diluted in MEM, and 100 µL of each serial dilution was inoculated into EPC cells in 96-well plate (1×10^4 cells per well). The plates were then placed in a 5% CO_2 cell incubator (Thermo) at 18 °C for 2 hours and replaced with 5% FBS/MEM. At 7 days post inoculation, the cells were inspected for signs of CPE via optical microscopy.

Immersion infection of fish with IHNV and sample collection. The fish were exposed to 2×10^4 $TCID_{50}$ IHNV per mL of water by immersion, and the mortality was recorded for 30 d. Two types of challenges with IHNV were performed to generate two groups of fish. For the first group, fish were exposed only once to the virus and then the SB tissue was collected at 1, 4, 7, and 14 DPI for the detection of viral load and pathological changes after fish were euthanized with an overdose of tricaine methanesulfonate (MS-222, Sigma). For the second group, fish surviving from the primary infection were challenged a second and a third time with the same dose of IHNV at 30 and 60 DPI, respectively. Thereafter, samples were collected at 75 DPI survivor fish (75DPI-S fish). Mock-infected (uninfected) fish were immersed with the same amount of culture medium from uninfected EPC cells as the control group.

Isolation of HK and SB leukocytes. To obtain leukocytes from HK and SB, the collected tissues were pressed through a 100-μm cell strainer (BD Biosciences) and suspended in a Dulbecco's modified eagle medium (DMEM, Thermo) supplemented with 1% FBS (Gibco) and 1% penicillin-streptomycin (Thermo). The cell suspensions from HK and SB were thereafter washed with the DMEM twice and were stored on ice until further use.

Flow cytometry analysis. Percentages of IgT^+ and IgM^+ B-cells in leukocytes of HK and SB and coating of Igs on SB microbiota were analyzed via flow cytometry as we previous described[3]. Briefly, the isolated leukocyte suspensions from the HK and SB were stained with mouse anti-trout IgT (clone 41.8; mouse IgG2b isotype; 1 μg/mL) and mouse anti-trout IgM (clone 1.14; mouse IgG1 isotype; 1 μg/mL) mAbs. Stained cells were detected with APC-conjugated goat anti-mouse IgG2b and PE-conjugated goat anti-mouse IgG1 (1 μg/mL each, BD Biosciences) pAbs. To detect the coating of Igs on SB microbiota, the bacteria suspension from SB mucus was stained with mouse anti-trout IgT, anti-trout IgM and anti-trout IgD (mouse IgG1 isotype) mAbs or their respective isotype controls (1 μg/mL), followed by a secondary staining with Alexa Fluor 488-conjugated goat anti-mouse IgG2b (for IgT detection, BD Biosciences) or Alexa Fluor 488-conjugated goat anti-mouse IgG1 (for IgM or IgD detection, BD Biosciences) antibodies, respectively. BacLight Red bacterial stain (Invitrogen) was used to discriminate bacteria from debris following the manufacturer's instructions. The analysis of stained leukocytes or bacteria was performed with a CytoFLEX flow cytometer (Beckman Coulter) and FlowJo software (FlowJo, LLC).

Histology, light microscopy, and immunofluorescence microscopy studies. The collected SB tissues were fixed in 4% neutral buffered formalin, dehydrated in graded ethanol, embedded in paraffin, sectioned with microtome (Thermo) at a thickness of 5 μm. Hematoxylin/eosin (H & E) stain and Alcian Blue-Periodic acid-schiff (AB-PAS) stain were performed as previously reported[4]. The detection of IgT^+ and IgM^+ B-cells in SB tissues was performed by immunofluorescence microscopy as we have described previously[5]. Briefly, the paraffin-section of SB was blocked by StartingBlock™ Blocking Buffer (Thermo) and then double-stained with rabbit anti-trout IgT pAb (Rabbit IgG isotype; 0.5 μg/mL) and mouse anti-trout IgM mAb (IgG1 isotype; 1 μg/mL) overnight at 4 °C. After washing three times with PBS, B-cells were detected by Alexa Fluor 488-conjugated AffiniPure Goat anti-rabbit IgG (for IgT detection) and Cy3-

conjugated AffiniPure Goat anti-mouse IgG (for IgM detection) (Jackson ImmunoResearch Laboratories Inc) at 2.5 μg/mL each for 40 min at room temperature (RT). To study the colocalization of trout pIgR and IgT in SB mucosa, the paraffin-section of SB was first stained with rabbit anti-trout IgT pAb (0.5 μg/mL) overnight at 4 °C, followed by Cy3-conjugated AffiniPure goat anti-rabbit IgG (2.5 μg/mL; Jackson ImmunoResearch Laboratories Inc) for 40 min at RT. After washing six times with PBS, the same paraffin-section was stained with the biotin-labeled anti-trout pIgR pAb (0.5 μg/mL) overnight at 4 °C, followed by the Alexa Fluor 488-conjugated Streptavidin (Jackson ImmunoResearch Laboratories Inc) for 40 min at RT. For the detection of IHNV infected cells in the SB tissue, the paraffin-sections were stained with mouse anti-IHNV-*N* mAb (Mouse IgG for isotype; 1 μg/mL; BIO-X Diagnostics) at 4 °C overnight. As controls, the mouse IgG was used at the same concentration. After washing three times with PBS, Cy3-conjugated AffiniPure goat anti-mouse IgG pAb (3 μg/mL) was added and incubated at RT for 40 min. All sections were stained with DAPI (4', 6-diamidino-2 phenylindole; 1 μg/mL; Invitrogen) for 8 min before mounting. For visualization of Ig coating on SB bacteria, the bacteria were first double-stained with rabbit anti-trout IgT and mouse anti-trout IgM (1 μg/mL each), or isotype controls (the rabbit IgG and the mouse-IgG1, 1 μg/mL each) at 4 °C for 2 h with continuous agitation. After washing three times with PBS, the secondary antibodies Alexa Fluor 488-conjugated AffiniPure Goat anti-rabbit IgG or Cy3-conjugated AffiniPure Goat anti-mouse IgG (Jackson ImmunoResearch Laboratories Inc.) were each added (2.5 μg/mL) and incubated for 30 min at 4 °C. After washing three times with PBS, biotin-labeled mouse anti-IgD antibody (1 μg/mL) was added and incubated at 4 °C for 2 h. SB bacteria were thereafter washed three times and Alexa Fluor 647-conjugated Streptavidin (Jackson ImmunoResearch Laboratories Inc.) was added (5 μg/mL) and incubated at 4 °C for 30 min. Before mounting, bacteria were stained with a mixed solution of DAPI and Hoechst 33342 dye (5 μg/mL; Molecular Probes). Stained bacteria were cytospinned on glass slides and mounted with fluorescent microscopy mounting solution. Images were captured using an Olympus BX53 fluorescence microscope and analyzed with the iVision-Mac scientific imaging processing software (Olympus).

SDS-PAGE and western blot analyses. The collected serum (1 μL) and SB mucus (40 μL) samples were kept in Laemmli sample buffer (Bio-Rad) and separated via 4-15% SDS-PAGE Ready Gel (Bio-Rad) under non-reducing and/or reducing conditions. Subsequently, the gels

were transferred onto Sequi-Blot polyvinylidene fluoride (PVDF) membranes (Bio-Rad) and blocked with 8% skim milk in PBS (pH 7.2) at RT for 2 h. The membranes were incubated with rabbit anti-trout IgT pAb, mouse anti-trout IgM or biotinylated mouse anti-trout IgD mAbs, followed by incubation with HRP-conjugated anti-rabbit IgG (for IgT detection, Abcam), anti-mouse IgG (for IgM detection, GE Healthcare) and Streptavidin (for IgD detection, Thermo), respectively[5]. Immunoreactive bands were visualized using the enhanced chemiluminescent reagent (Advansta) and scanned by an Amersham Imager 600 imaging system (GE Healthcare). For quantitative analysis of IgT, IgM, and IgD in serum and SB mucus, the signal strength of each band was determined by using ImageQuant TL software (GE Healthcare). Thereafter, the concentration of IgT, IgM and IgD were determined by plotting the obtained signal strength values on a standard curve generated for each blot using known amounts of purified trout IgT, IgM and IgD, respectively, as previously described by us[5]. Original images of the western blot analyses are shown in Supplementary Fig. S11.

Gel filtration. Gel filtration was conducted to analyze the monomeric or polymeric state of Igs in trout SB mucus using a Superdex-200 FPLC column (GE Healthcare) as previously described by us[5]. Western blot analysis was performed to identify IgM, IgD, and IgT in the eluted fractions using IgM-, IgD-, and IgT-specific antibodies.

Co-immunoprecipitation studies. To evaluate whether the polymeric IgT present in the trout SB was associated with trout secretory component (tSC) co-immunoprecipitation studies were performed to detect the association of trout pIgR (i.e., tSC) to IgT in the SB, as described in our previous study[3]. We performed co-immunoprecipitation analysis using rabbit anti-trout IgT pAb with the goal to co-immunoprecipitate tSC. To this end, 10 μg of anti-trout IgT pAb were incubated with 300 μL of trout SB mucus. As control for these studies, the same amount of rabbit IgG (purified from the pre-bleed serum of the rabbit) were used as negative controls. After overnight incubation at 4 °C, 20 μL of Dynabeads protein G (Invitrogen) prepared previously was added into each reaction mixture and incubated for 1 h at 4 °C according to the manufacturer's instructions. Thereafter, the beads were washed five times with PBS, and then, the subsequently bound proteins were eluted in 2 × Laemmli Sample Buffer (Bio-Rad). The eluted material was resolved on 4-15% SDS-PAGE Ready Gel under reducing (for tSC detection) or non-reducing (for IgT detection) conditions. Western blot was performed with

rabbit anti-trout pIgR and rabbit anti-trout IgT pAbs as described above.

RNA isolation and qPCR analysis. Total RNA from each sample was extracted using the TRIzol reagent (Invitrogen) according to the manufacturer's protocols and resuspended in RNase-free H_2O. The concentration and integrity of RNA were respectively detected by spectrophotometry (Implen NanoPhotometer NP 80 Touch, Germany) and agarose gel electrophoresis. Thereafter, equivalent amounts of the total RNA (1 µg) were reverse transcribed using Hifair III 1st Strand cDNA Synthesis SuperMix for qPCR (gDNA digester plus) (YEASEN). The synthesized cDNA was diluted four times and then used as a template for qPCR analysis. qPCR was conducted using the MonAmp^{TM} SYBR Green qPCR Mix (Monad) following the manufacturer's instructions. Viral loads were determined by constructing an IHNV plasmid standard curve, average values from duplicates of each gene in the samples were extrapolated using the standard curve to calculate the IHNV copy numbers. Supplementary Table 1 summarizes the primers used for qPCR analysis.

Viability of infected EPC cells and expression of IHNV in cells. The potential effects of IHNV on EPC cell viability were analyzed by the alamarBlue assay using alamarBlue™ Cell Viability Reagent (Invitrogen) according to the manufacturer’s instructions. Briefly, 1/10th volume of cell viability reagent was directly added to the medium of IHNV-infected EPC cells in 96-well plates and incubated for 4 h at 18 °C in a cell culture incubator. The absorbance of alamarBlue was measured at a wavelength of 570 nm using a SpectroStar Omega absorbance microplate reader (BMG LabTech). The background absorbance was measured at 600 nm and subtracted. The percent reduction of alamarBlue was calculated using the manufacturer's instructions[6,7]. Detection of IHNV protein *N* in infected EPC cells was performed by immunofluorescence and western blot analyses with mouse anti-IHNV-*N* mAb (Bio-X Diagnostics). For the immunofluorescence microscopy of IHNV in EPC cells, the infected cells were fixed in 4% neutral buffered formalin and stained with mouse anti-IHNV-*N* mAb (1 µg/mL) at 4 °C overnight and then stained with Cy3-conjugated AffiniPure goat anti-mouse IgG pAb (3 µg/mL) at room temperature for 40 min. Images were captured using the inverted microscope (Leica). To detect IHNV by western blotting, the infected cells were collected and lysed with Cell Lysis Buffer (Beyotime) at a ratio of 2×10^4 cells to 100 µL of lysis buffer. The cell lysate was centrifuged at 12, 000 *g* for 10 min at 4 °C to remove particle debris. Thereafter, 20 µL of the

resulting supernatant was mixed with 20 μL 2 × Laemmli sample buffer, proteins were separated via 4-15% SDS-PAGE Ready Gel under reducing conditions, and detected by mouse anti-IHNV-*N* mAb (0.2 μg/mL) for western blot.

RNA-Seq library construction and bioinformatic analyses. For the construction of RNA-seq libraries, twelve SB tissue samples (including control and IHNV infection groups at 4 and 21 DPI) were obtained and total mRNA was extracted using the TRIzol reagent and resuspended in RNase-free H_2O. Thereafter, mRNA was purified from the total RNA using poly-T oligo-attached magnetic beads (Thermo) following the manufacturer's instructions. Short mRNA fragments were reverse transcribed into first-strand cDNA using random hexamer (Thermo) and Superscript II reverse transcriptase (Thermo), followed by the synthesis of second-strand cDNA using RNaseH (QIAGEN) and DNA polymerase I (QIAGEN). The 3′ ends of the DNA fragments were adenylated and a unique identifier (UID) was added at the 5′ ends before ligating them to Illumina paired-end sequencing adaptors. PCR amplification was conducted using the Phusion High-Fidelity Master Mix Phusion High-Fidelity Master Mix (Thermo) and Illumina primers Phusion High-Fidelity Master Mix (Thermo) according to the manufacturer's instructions. All RNA-seq data were generated by Illumina paired-end sequencing with 150 bp read lengths. Reads were mapped to the *Oncorhynchus mykiss* genome using STAR with default parameters[8]. The mapped reads were analyzed via feature counts[9]. Differential expression was estimated with the edgeR package[10]. Genes with low expression levels (count-per-million <1 in three or more samples) were excluded from downstream analyses. The resulting genes were considered differentially expressed genes (DEGs) if the false discovery rate ≤0.05 and $|\log_2$ (fold-change) $| \geq 1$. To further understand the transcriptomic data, gene ontology (GO) enrichment analysis was conducted using Kobas to determine the biological processes that were significantly dysregulated following IHNV infection[11].

Behavioral assay. The behavioral assay of rainbow trout was performed as previously described[12]. Briefly, each fish was placed in a ~6.6 L glass tank (26 cm × 18 cm × 14 cm). The locomotor trajectory was monitored using a ZebraTower system (ViewPoint). Movements were recorded every 5 s over a 5 min period and raw data was transferred into behavioral parameters that were conducted by Zebralab (Viewpoint).

Tissue explants culture. HK and SB were collected from the control and survivor fish, and the explants were cultured as previously described by us[5]. Briefly, tissues were submerged in 70% ethanol for 30 s to eliminate contaminating bacteria on the surface and then washed twice with PBS. Thereafter, tissues were placed in a 24-well plate and cultured with 200 μL DMEM (Gibco), supplemented with 10% FBS, 100 μg/mL streptomycin, and 100 U/mL penicillin, with 5% CO_2 at 18 °C. After 7 days culture, supernatants were harvested, centrifuged, and stored at -80 °C for detection of IgM-, IgT-, and IgD-specific binding to IHNV.

Proliferation of B-cells in the SB of trout. Survivor and control fish (~10 g) were anaesthetized with MS-222 and intravenously injected with 200 μg 5-ethynyl-2'-deoxyuridine (EdU, Invitrogen) in 100 μL PBS. After 24 hours, SB tissues were obtained, and the proliferation of EdU^+ cells were detected by immunofluorescence microscopy and flow cytometry analyses as previously described by us[5]. After the staining of IgT^+ and IgM^+ B-cells described above, stained cells were fixed and EdU^+ cell detection was performed by Click-iT EdU Alexa Fluor 647 Imaging Assay Kit (for imaging) and Click-iT EdU Alexa Fluor 488 Flow Cytometry Assay Kit (for flow cytometry) (Invitrogen) according to the manufacturer's instructions. For immunofluorescence analysis, cell nuclei were stained with DAPI (1 μg/mL) before mounting with fluorescent microscopy mounting solution. The images were acquired and analysed by using BX53 fluorescence microscope (Olympus) and iVision-Mac scientific imaging processing software (Olympus). For flow cytometry analysis, cells were analysed with a CytoFLEX Flow Cytometer (Beckman Coulter) and FlowJo software (FlowJo, LLC).

Supplementary References

1. Liu, H., Liu, Y., Liu, S., Pang, D.W. & Xiao, G. Clathrin-mediated endocytosis in living host cells visualized through quantum dot labeling of infectious hematopoietic necrosis virus. *J. Virol.* **85**, 6252-6262 (2011).
2. Reed, L.J. & Muench, H. A simple method of estimating fifty per cent endpoints. *Am. J. Epidemiol*. **27**, 493-497 (1938).
3. Yu, Y.Y. et al. Mucosal immunoglobulins protect the olfactory organ of teleost fish against parasitic infection. *PLoS. Pathog*. **14**, e1007251 (2018).
4. Crespo-Moral, M., García-Posadas, L., López-García, A. & Diebold, Y. Histological and immunohistochemical characterization of the porcine ocular surface. *PLoS One* **15**, e0227732 (2020).
5. Xu, Z. et al. Mucosal immunoglobulins at respiratory surfaces mark an ancient association that predates the emergence of tetrapods. *Nat. Commun.* **7**, 10728 (2016).
6. Pettit, R.K., Weber, C.A. & Pettit, G.R. Application of a high throughput Alamar blue biofilm susceptibility assay to Staphylococcus aureus biofilms. *Ann. Clin. Microbiol. Antimicrob.* **8**, 28 (2009).
7. Byth, H.A., McHunu, B.I., Dubery, I.A. & Bornman, L. Assessment of a simple, non-toxic Alamar blue cell survival assay to monitor tomato cell viability. *Phytochem. Anal*. **12**, 340-346 (2001).
8. Dobin, A. et al. STAR: ultrafast universal RNA-seq aligner. *Bioinformatics*. **29**, 15–21 (2013).
9. Liao, Y., Smyth, G.K. & Shi, W. Feature Counts: an efficient general purpose program for assigning sequence reads to genomic features. *Bioinformatics*. **30**, 923–930 (2014).
10. Robinson, M.D., McCarthy, D.J. & Smyth, G.K. edgeR: a Bioconductor package for differential expression analysis of digital gene expression data. *Bioinformatics* **26**, 139–140 (2010).
11. Wu, J., Mao, X., Cai, T., Luo, J. & Wei, L. KOBAS server: a web-based platform for automated annotation and pathway identification. *Nucleic Acids Res.* **34**, 720–724 (2006).
12. Shi, C. et al. Hyperandrogenism in POMCa-deficient zebrafish enhances somatic growth without increasing adiposity. *J. Mol. Cell Biol.* **12**, 291–304 (2020).

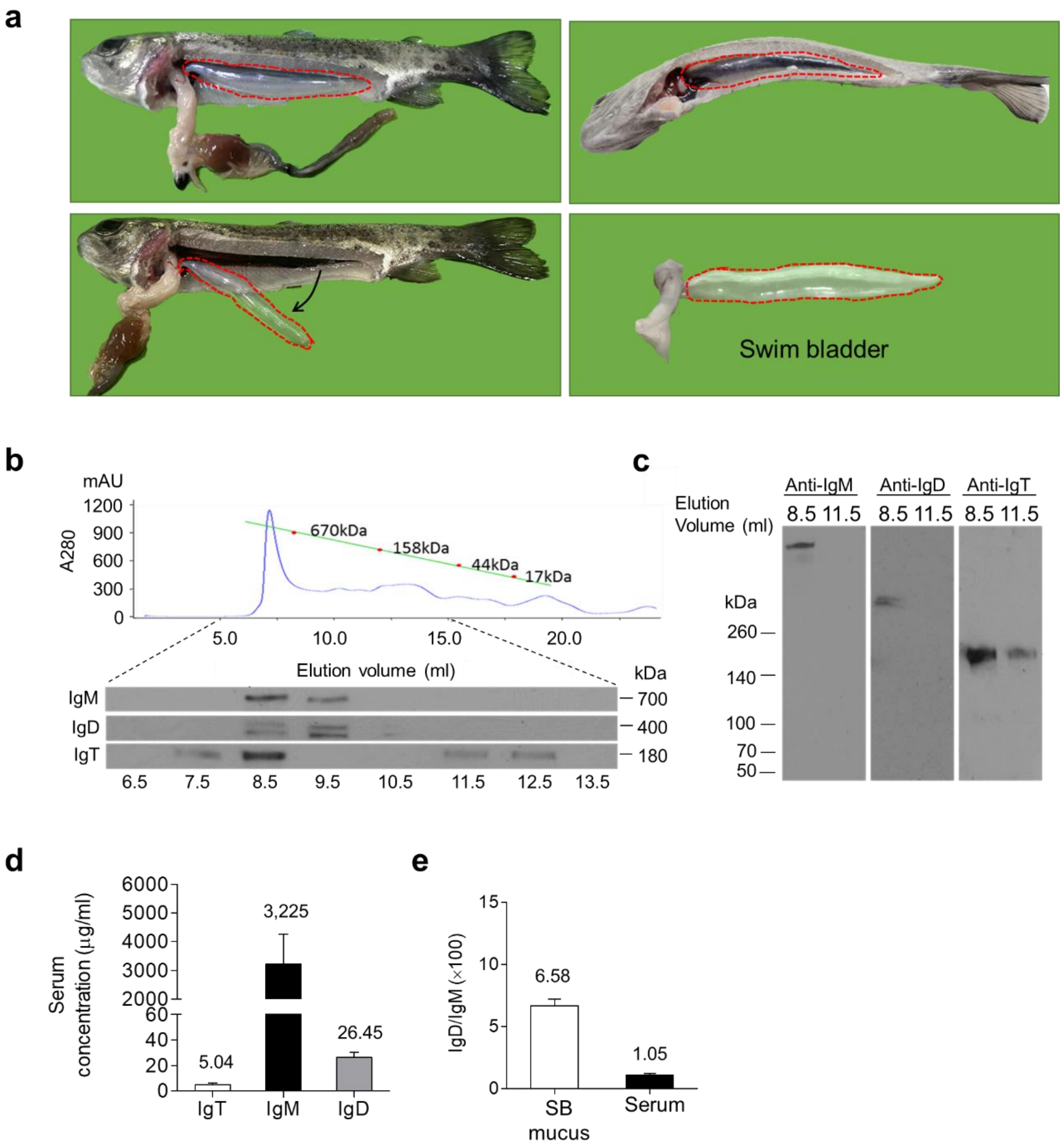

Supplementary Fig. S1 The gross anatomy and location of SB in trout, and the protein characterization of SB mucus Igs. **a** Gross anatomy and location of SB in trout. **b** Fractionation of SB mucus (~0.5 mL) by gel filtration (upper) followed by immunoblot analysis of the eluted fractions (elution volumes 6.5-13.5 mL) with anti-trout IgM and anti-trout IgD mAbs or anti-trout IgT antibody (lower). A_{280}, absorbance at 280 nm. **c** SDS-PAGE (4-15%) of gel-filtration fractions corresponding to eluent at elution volumes of 8.5 mL and 11.5 mL under non-reducing conditions, followed by immunoblot analysis with anti-trout IgM and anti-trout IgD mAbs or

anti-trout IgT pAb. **d** Concentration of IgT, IgM, and IgD in serum (*n* = 13). **e** Ratio of IgD to IgM concentration in SB mucus and serum (*n* = 13).

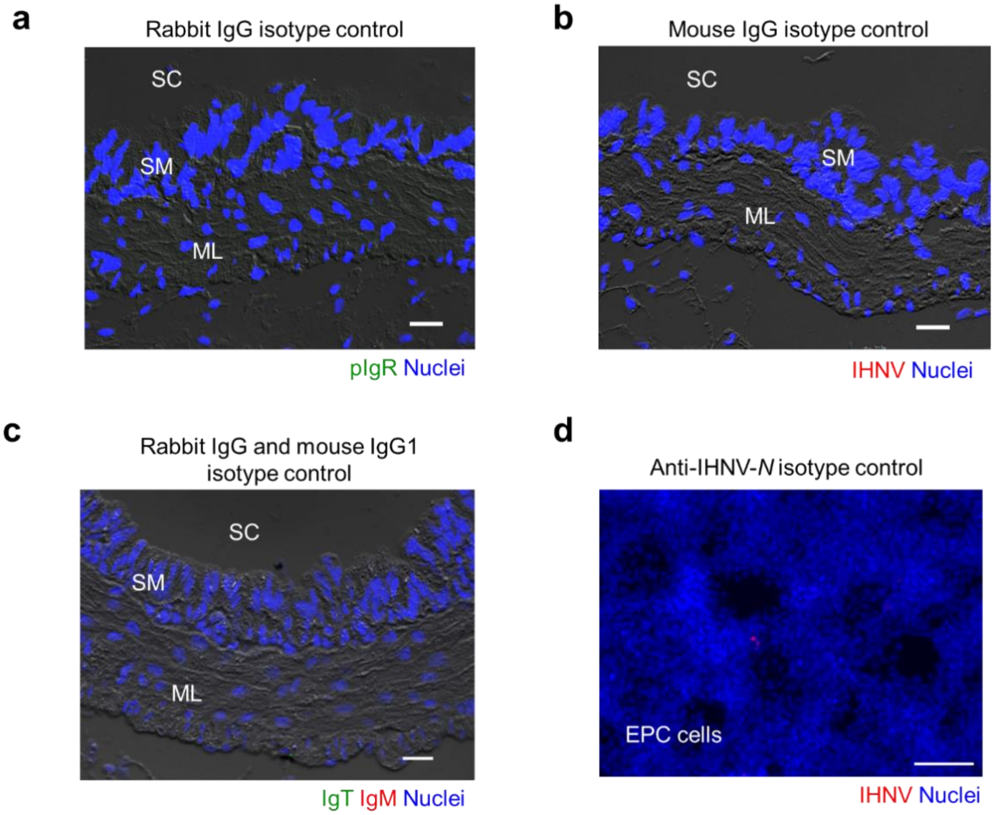

Supplementary Fig. S2 Isotype control staining for anti-pIgR, anti-IHNV-*N*, anti-IgT and anti-IgM, antibodies in trout SB paraffin sections, and isotype control staining for anti-IHNV-*N* antibody in EPC cells. a-c DIC images of SB paraffin-sections, with merged staining of isotype control antibodies for anti-trout pIgR pAb (green, **a**), anti-IHNV-*N* mAb (magenta, **b**) and anti-trout IgT pAb (green, **c**) and anti-trout IgM mAb (red, **c**). **d** IHNV infected EPC cells merged staining of isotype control antibodies for anti-IHNV-*N* mAb (magenta, **d**). Nuclei were stained with DAPI (blue) (**a-d**). SC, SB cavity; SM, SB mucosa; ML, muscle layer. Scale bars, 20 μm. Data are representative of three independent experiments.

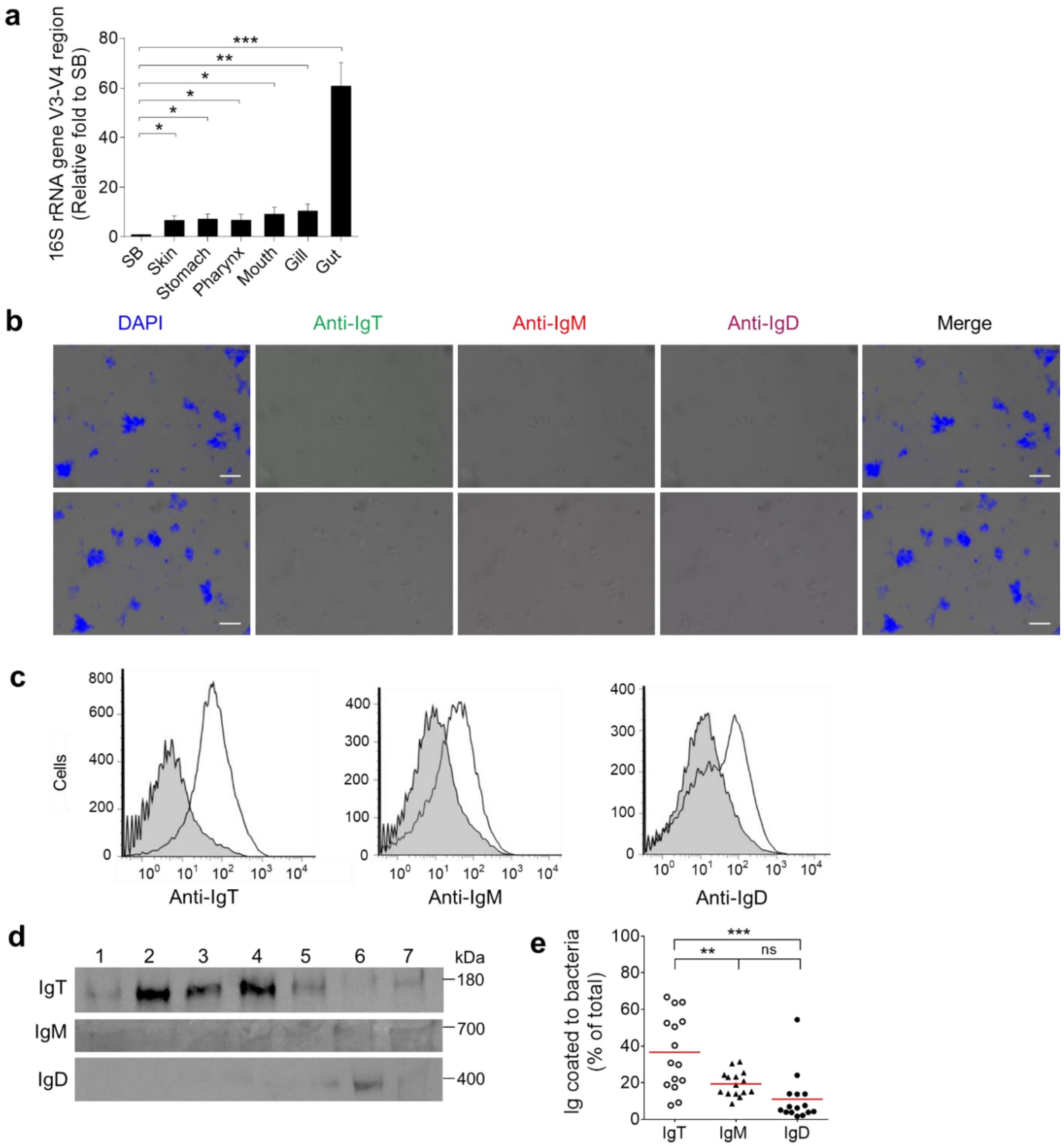

Supplementary Fig. S3 Presence of microbiota in SB and analysis of their coating by trout sIgs. a Real-time PCR analysis of bacteria V3-V4 16S rRNA region in different trout mucosal tissues (SB, skin, stomach, pharynx, mouth, gill, and gut). DNA abundance was normalized to that of SB, which is set as 1 (n = 6). **b** Differential interference contrast of SB bacteria with isotype control antibodies for anti-trout IgT (green), anti-trout IgM (red), or anti-trout IgD (magenta) mAbs. Nuclei were stained with DAPI (blue). Scale bars, 5 μm. Upper and lower panels display two different samples representative of at least three independent experiments. **c**

Representative flow cytometry histograms showing the staining of SB bacteria with anti-trout IgT, IgM, and IgD mAbs. Bacteria were stained also with isotype control antibodies for anti-trout IgT, anti-trout IgM, or anti-trout IgD mAbs, respectively (shaded histograms). **d** Immunoblot analysis of IgT, IgM, and IgD on SB bacteria ($n = 7$). **e** Percentage of total SB mucus IgT, IgM, or IgD coating the SB bacteria ($n = 15$). The median value is shown by a red line. Statistical differences were evaluated by unpaired Student's *t*-test (**a**) and one-way ANOVA with Bonferroni correction (**e**). Data in **a** and **e** are representative of at least three independent experiments (mean ± SEM). $^{*}P < 0.05$, $^{**}P < 0.01$, and $^{***}P < 0.001$.

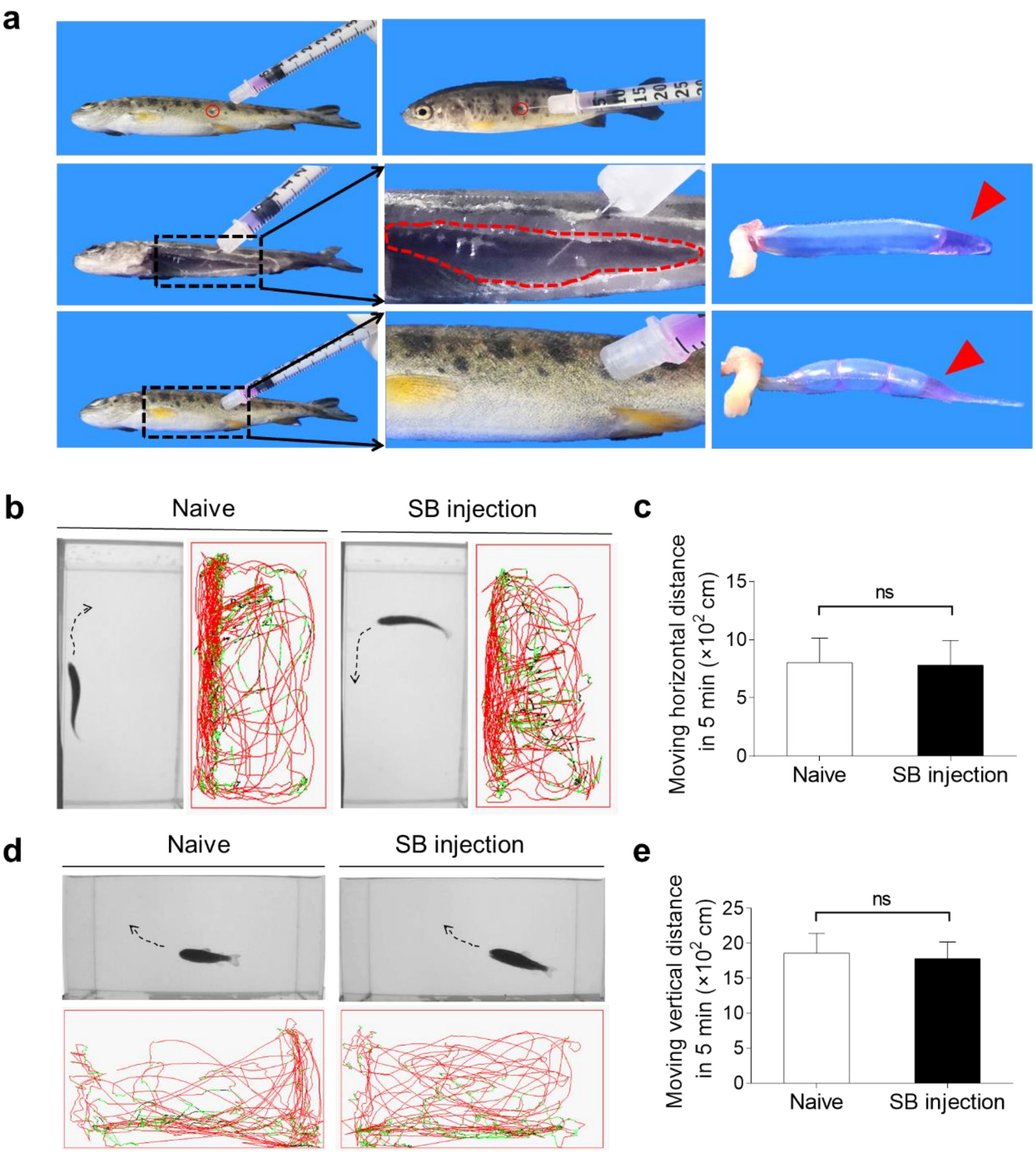

Supplementary Fig. S4 Delivery of medium solution into SB lumen does not affect swimming behavior of SB-injected fish. a DMEM is effectively delivered into the SB without affecting the air volume of the SB. The empty red circles represent the injection position; the red dashed line shows the SB containing the syringe needle; the red arrow represents DMEM delivered into the SB. **b** Horizontal locomotor trajectory over a 5 min period of trout with or without SB injection with DMEM. The movement velocities >4.5 cm s^{-1} and <2.5 cm s^{-1} are presented by red and gray locomotor trajectories respectively while the movement velocity between 2.5 cm s^{-1} and 4.5 cm s^{-1} is represented by a green locomotor trajectory. **c** Total horizontal distance moved over 5 min by trout with and without SB injection (*n* = 11-12). **d**

Vertical locomotor trajectory of trout with and without SB injection with DMEM over a 5 min period. **e** Total horizontal distance moved over 5 min by trout with or without SB injection (*n* = 4-5). Statistical analysis was performed by unpaired Student's *t*-test (**c** and **e**). Data in **c** and **e** are representative of at least three independent experiments (mean ± SEM). ns, no significance.

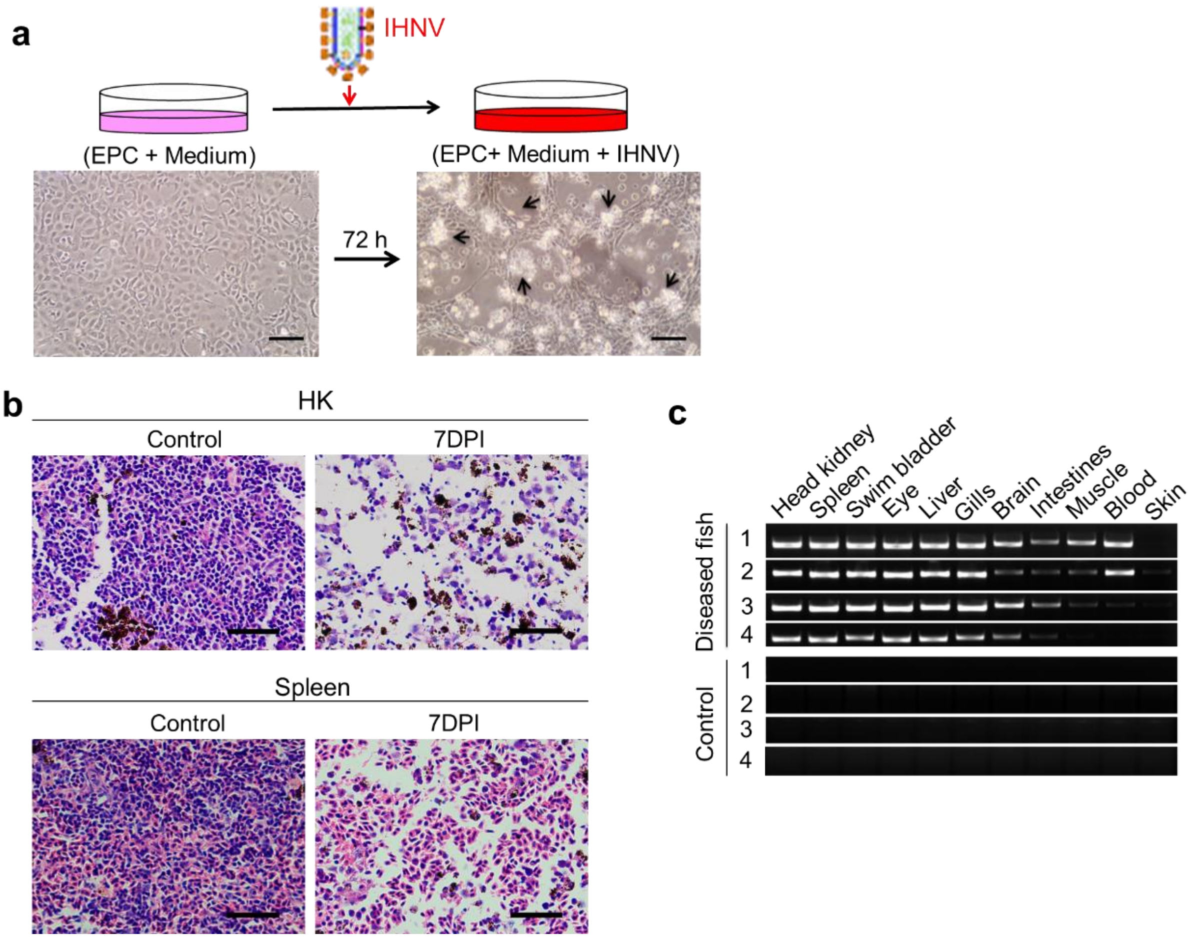

Supplementary Fig. S5 Cytopathic effects of IHNV in EPC cells, HK and spleen, and IHNV load in different organs of infected trout. a Cytopathic effect shown in the EPC cells at 72 h after IHNV infection (small black arrows show cytopathic lesions). Scale bars, 50 μm. **b** H & E staining of HK and spleen paraffin-sections in control (left) and infected (right) fish after 7 DPI with IHNV (25 μL, 1×10^5 $TCID_{50}$). Scale bars, 50 μm. **c** IHNV-*G* gene (693 bp) expression profiles by reverse transcription PCR in different tissues from control and diseased trout, respectively.

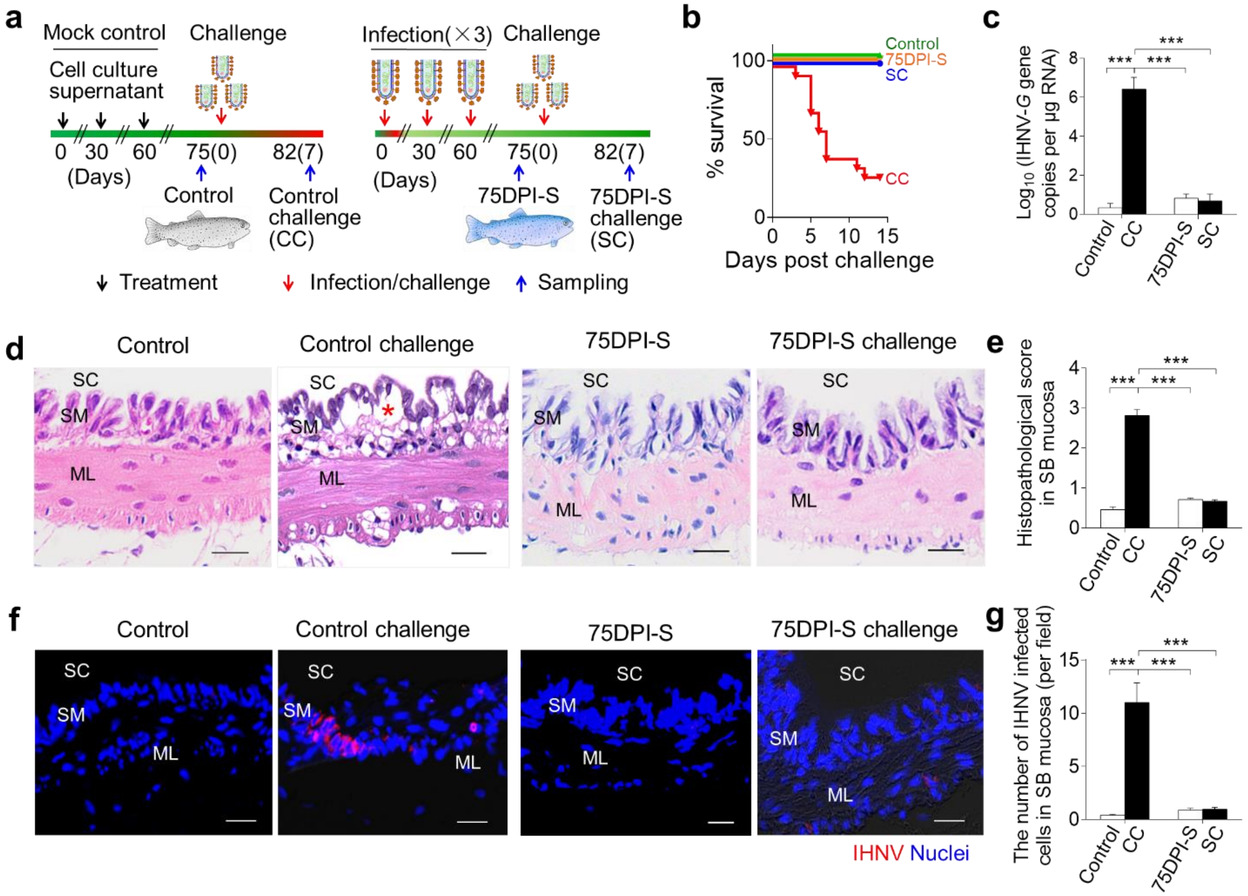

Supplementary Fig. S6 Survivor fish previously infected with IHNV become resistant to infection upon re-challenge with a high dose of the virus. a Strategy to obtain the different groups of control and survivor fish. Control fish were obtained after three monthly SB injections with EPC cells culture supernatant. At 75 days after the first injection, fish were challenged with the virus (25 µL, 1×10^6 $TCID_{50}$), and 7 days post-challenge fish (control challenge or CC group) were sacrificed for sampling. To generate 75 DPI survivor fish (75DPI-S group), fish were injected monthly with a viral preparation (25 µL, 1×10^5 $TCID_{50}$), and at 75 days after the first injection, fish were challenged with IHNV (25 µL, 1×10^6 $TCID_{50}$), and 7 days post-challenge fish (75DPI-S challenge or SC group) were sacrificed for sampling. **b** Percentage survival of control, CC, 75DPI-S and SC fish groups. **c** The IHNV-*G* gene expression in SB of control, CC, 75DPI-S and SC fish groups were quantified using qPCR. **d** Histological examination by H & E staining of SB from control, CC, 75DPI-S and SC fish (n = 7-9). Red asterisk indicates tissue damage in epithelium layer of SB. SC, SB cavity; SM, SB mucosa; ML, muscle layer. Scale bars, 20 µm. **e** Pathology score of SB tissue from control, CC, 75DPI-S and SC fish were evaluated on

SB tissue sections stained with H & E. **f** Viral particles were detected with the anti-IHNV-*N* protein mAb (red) in SB mucosa from control, CC, 75DPI-S and SC fish (n = 8-12). SC, SB cavity; SM, SB mucosa; ML, muscle layer. Scale bars, 20 μm. **g** The number of virally stained cells in SB mucosa from control, CC, 75DPI-S and SC fish counted from **f** (n = 8-12). Statistical analysis was performed by unpaired Student's *t*-test (**c**, **e**, **g**). Data are representative of at least three independent experiments (mean ± SEM). ***P < 0.001.

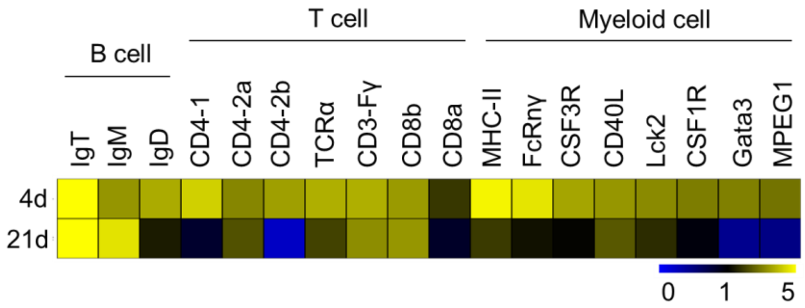

Supplementary Fig. S7 Increases in the mRNA expression levels of B cell, T cell and myeloid markers in trout SB upon infection with IHNV. Heat map illustrates results from quantitative real-time PCR of transcripts for selected immune markers from SB of IHNV-infected versus control fish measured at 4 and 21 DPI (n = 6). Data are expressed as mean fold increase in expression. Color value: $\log_2$ (fold change).

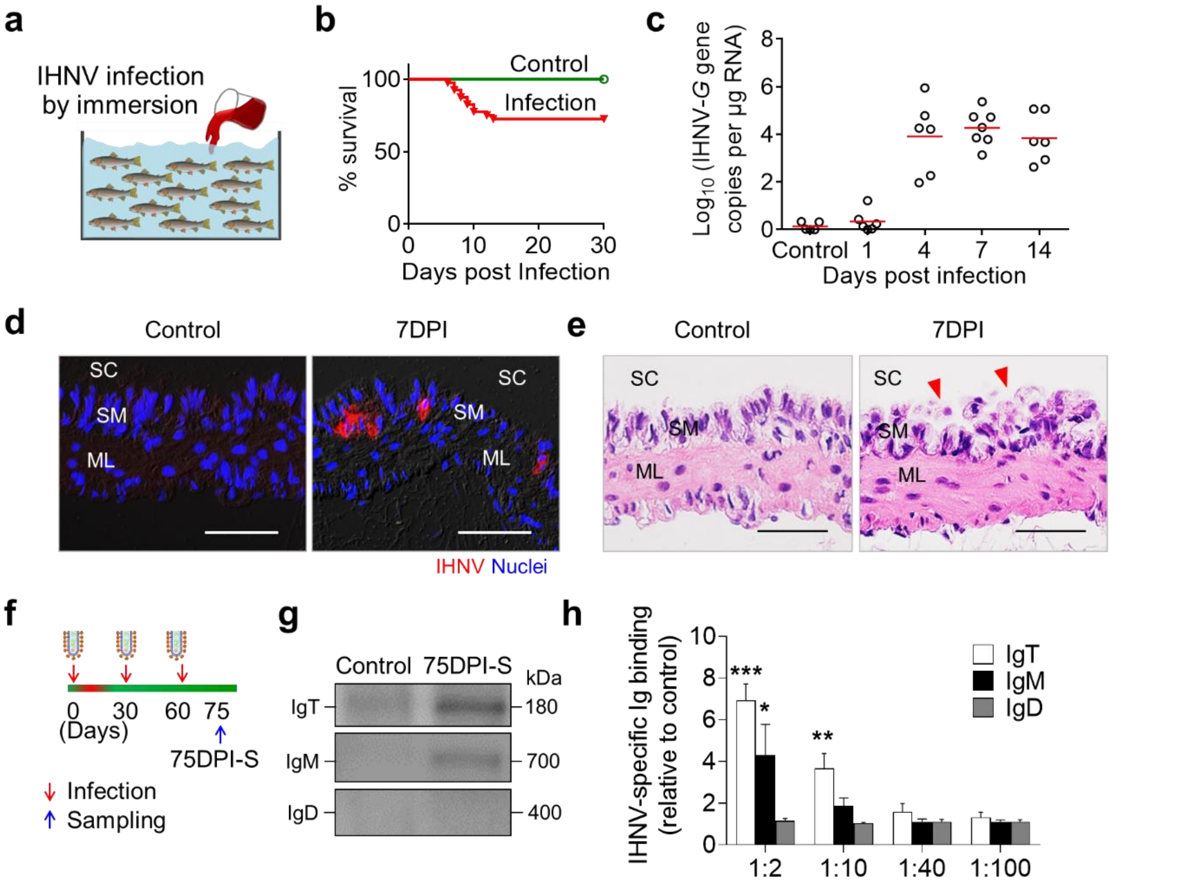

Supplementary Fig. S8 The Ig response of trout SB following infection by immersion with IHNV. a Trout were infected by immersion with a dose of IHNV (2×10^4 $TCID_{50}$ per mL of water), and treated with medium as control. **b** Cumulative survival of control and IHNV-infected fish (*n* = 40 per group). **c** IHNV-*G* gene copies (Log_{10}) were quantified using qPCR in fish tissues collected at 1, 4, 7, and 14 DPI (*n* = 6). The median value is shown by a red line. **d** Immunofluorescence staining of IHNV in SB paraffin-sections from control and 7 days infected fish (*n* = 6). **e** Histological examination (H & E) of SB from control fish and 7 days infected fish. The red arrow represents the damage of SB epitheliums upon IHNV infection. **f** fish were infected by immersion with the same dose of IHNV. The surviving fish were reinfected twice at 30 and 60 DPI with the same strategy, and the resulting surviving fish were sacrificed at 75 days after the first infection (75DPI-S group). **g** Western blot analysis of IgT-, IgM- and IgD-specific binding to IHNV in SB mucus (dilution 1/2) from 75DPI-S fish comparted with the control. **h** IgT-, IgM- and IgD-specific binding to IHNV in dilutions of SB mucus from 75DPI-S fish

evaluated by densitometric analysis of immunoblots and presented as relative values to those of control fish ($n = 6$).

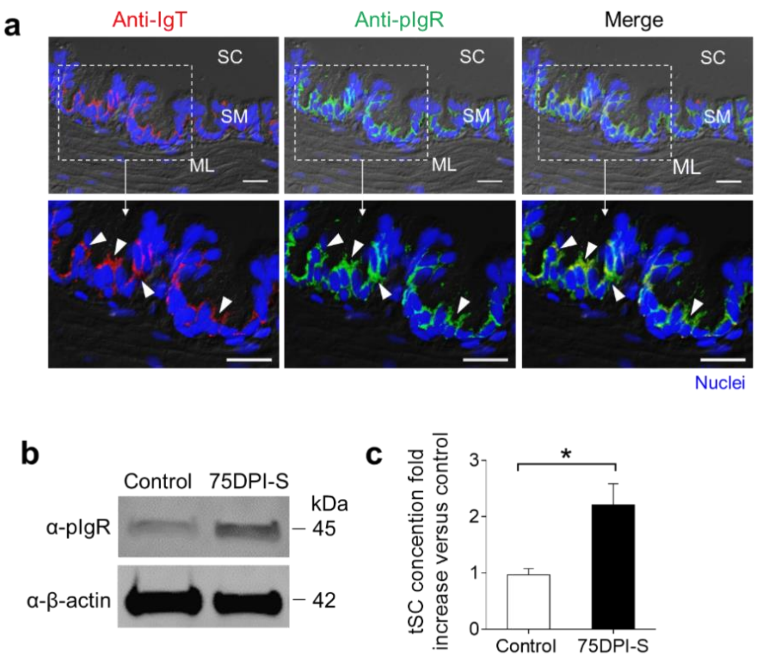

Supplementary Fig. S9 Trout pIgR associates with sIgT in SB mucosa and pIgR levels are increased in 75DPI-S fish. a Immunofluorescence staining for pIgR and IgT in SB mucosa of 75DPI-S trout. Differential interference contrast (DIC) images of SB mucosa stained with anti-trout IgT (red), anti-trout pIgR (green), and DAPI for nuclei (blue) (*n* = 6). Enlarged sections (**a**, lower panels) of the areas outlined in **a** (upper panels) showing pIgR/IgT colocalization (white arrowheads). Scale bars, 20 μm. SC, SB cavity; SM, SB mucosa; ML, muscle layer. **b** Representative immunoblot analysis of trout secretory component (tSC) of pIgR (upper panels) in SB mucus from control fish and 75DPI-S fish. **c** Data in B were quantified by densitometry (*n* = 5-7). Statistical difference in **c** was evaluated by unpaired Student's *t*-test. Data are representative of at least three independent experiments. *$P < 0.05$.

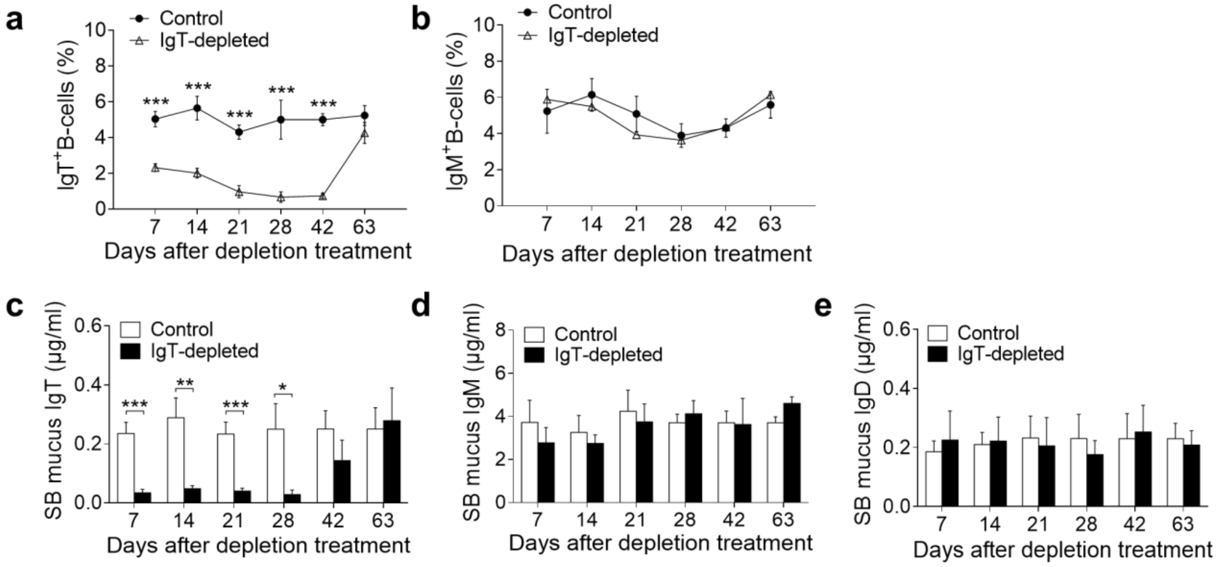

Supplementary Fig. S10 Effect of IgT+ B-cell depletion treatment on B-cells and sIgs in trout SB. a, **b** The percentage of IgT+ (**a**) and IgM+ (**b**) B-cells from SB leukocytes in control (non-depleted) and IgT+ B-cell-depleted fish (*n* = 7-9). **c-e** Concentration of IgT (**c**), IgM (**d**), and IgD (**e**) in SB mucus of control and IgT+ B-cell-depleted fish (*n* = 6). Data are representative of at least three independent experiments (mean ± SEM). Statistical differences were evaluated by unpaired Student's *t*-test. Data are representative of three independent experiments (mean ± SEM). *$P < 0.05$, **$P < 0.01$, and ***$P < 0.001$.

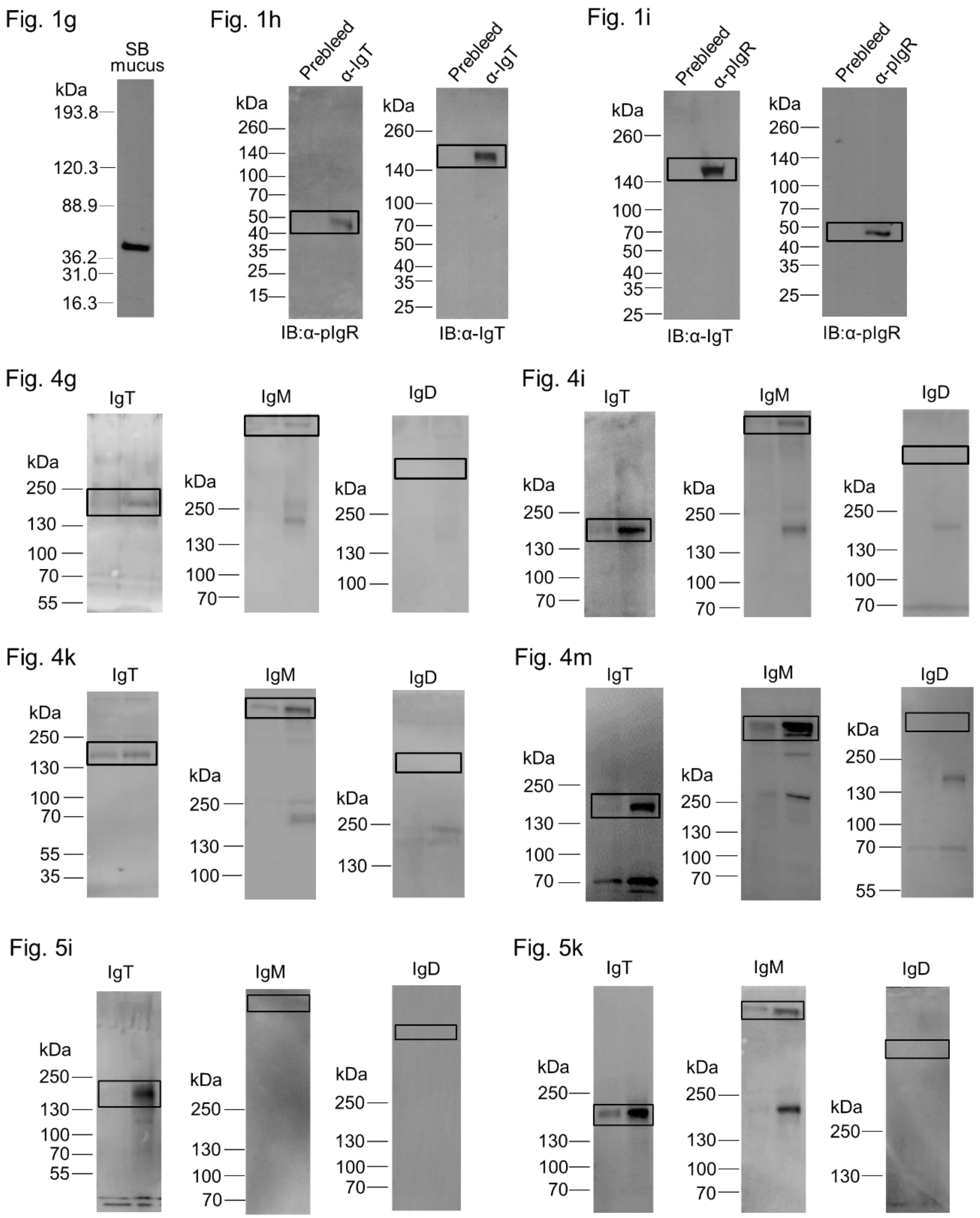

Supplementary Fig. S11 Original images of the western blot analyses in the main figures.

Fig. 6c

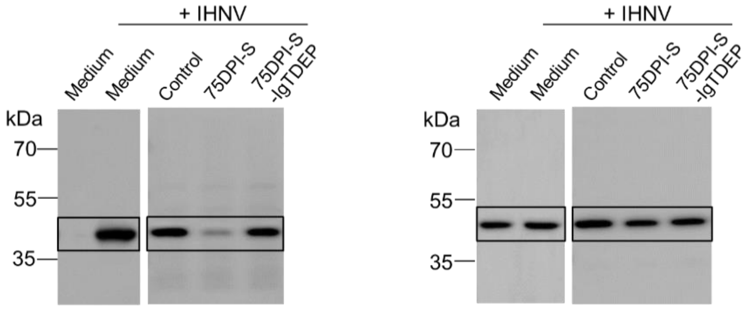

Supplementary Fig. S1b

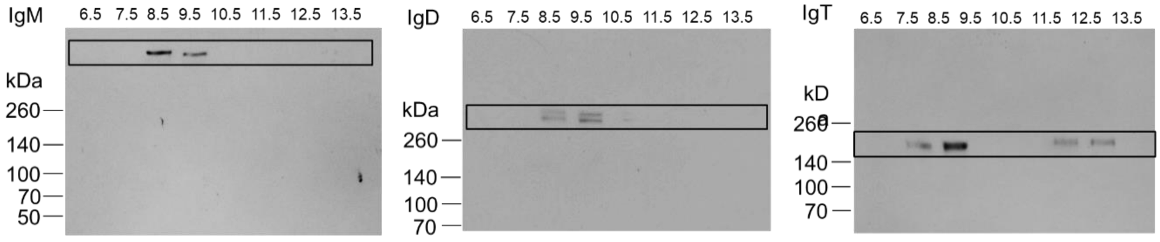

Supplementary Fig. S1c

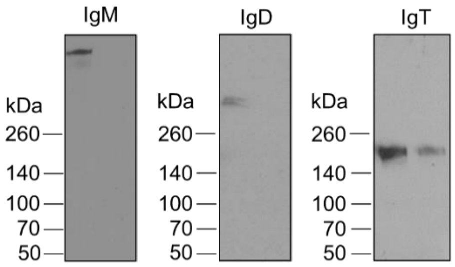

Supplementary Fig. S3d

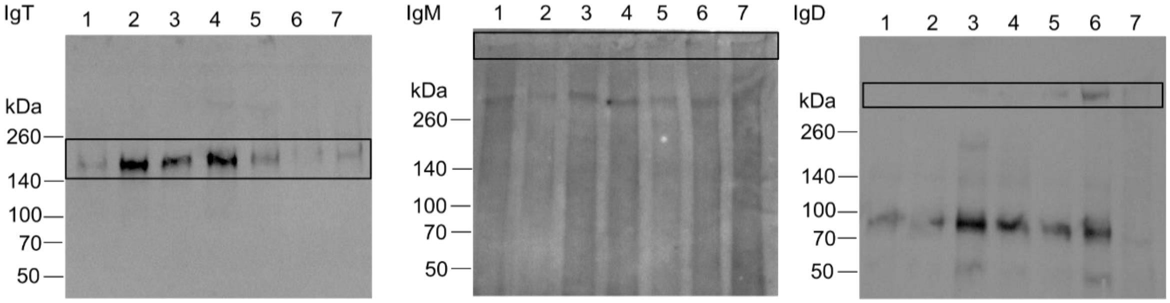

Supplementary Fig. S8g

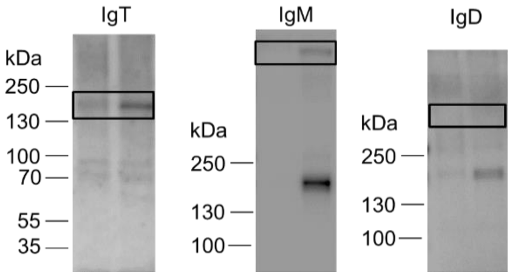

Supplementary Fig. S9b

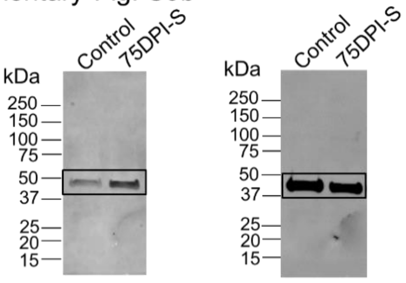

Supplementary Fig. S11 Continued

Supplementary Table. S1 Primers used in this study.

Gene	Primer Sequence (5’-> 3’)	
	Forward primer	Reverse primer
RIG-I	CAGAGGTACTACAGGAAATGG	TTACTGGTCTTCAAGCAATG
IRF7	GATGCCTCAAAATGAAATGG	TCAGGGAACTTCTCACCAAA
Mx1	GATGCTGCACCTCAAGTCCTACTA	CGGATCACCATGGGAATCTGA
CD80	ACACCGTTGTGAAAACTCCA	TCGTGCCCAATACAATAGAA
ITAC	GCCAGGTGGGTCATTCTAAA	TTCTTCCCTTCTCCGTTCTT
CD2	GGCAACCACGACTGGGACT	TGGCGTCTTTGAAGGTTTATT
IgT	CAGACAACAGCACCTCACCTA	GAGTCAATAAGAAGACACAACGA
IgM	AAGAAAGCCTACAAGAGGGAGA	CGTCAACAAGCCAAGCCACTA
IgD	CAGGAGGAAAGTTCGGCATCA	CCTCAAGGAGCTCTGGTTTGGA
PI3K	GAATAAGCACGAGGATGTA	CAGAAAAGTAGCAAGGTCA
CD4L	TTGACCTGGTAGCAAAAGC	TGAATGTGACTGTGATGGG
CXCL9	GTGGTTTTGCTGGGAGTTT	TTTTGCTTGTCGTCCTTGT
CXCL11	CCAGGTGGGTCATTCTAAA	CACTTCTTCCCTTCTCCGT
LGP2	AGTTTGGCACGCAGGAGTA	CAAGCAGGAAGAAGTCGGT
MDA5	CAGTGGAGATGACGATGGG	ACTTGGCGTTCTTGTGCTT
CD4-1	TGGTCGAGAGACGATAGATCC	GAGGTACTTGTTTGTGGCATGA
CD4-2a	CGTGAGAAGTTTGTTGCCGAA	TGGCTGCCTTTGGTACAGTGA
CD4-2b	AAGCCCCTCTTGCCGAGGAA	CTCAACGCCTTTGGTACAGTGA
TCR-α	CAGCTTGAAGTCAAGAAATAC	TATCAGCACGTTGAAAACGAT
CD3-Fγ	GAACACTGGAATACAAGGACGAGAACAC	GAGCCCCATTTTGCTAGATGTTTTCTT
CD8a	ACTGCCAAGTCGTGCAAAGTG	AAGCCACAGCCAGCAGTCAA
CD8b	TCCTGTATGCTCCAGAACCAG	ATGTTGGGCGAGTTTCTCCG
MHC-II	GGTGAGTTTGTTGGATAC	AGCGTTAGGCTTACATAGA
FcRnγ	TACTCCAACTCTCCATCTACTC	CTGTGGATACCCGCCAGTGA
CSF3R	TCCACGGGACAGAGTACCACA	GAAACTGCTTCGATGGCTTCC
CD40L	CAAGCAACCTGTCGTTGGTG	GTACACACGTCTGTCCGGTT
Lck2	CCTGTTGAAGAGCATTATATTAG	ACGGTTTAGCCGACTGGGTG
CSF1R	ATCTCCACTCATGGCGACACA	CATCGCACTGGGTTTCTGGTA
Gata3	CCAAAAACAAGGTCATGTTCAGAAGG	TGGTGAGAGGTCGGTTGATATTGTG
MPEG1	CTCAGACGTGTCCTTCCTCTC	CGTGTATAAGAAGTTACGCACTTG
EF-1a	CAACGATATCCGTCGTGGCA	CAACGATATCCGTCGTGGCA
IHNV-N	TTAACTTCAACGCCAACAGG	TCGGACAGGTTGATGAGAATG
IHNV-G	CACGGAAACAACACCACCATTA	AACAGCAAGGAGGAGAACAAGG
IHNV-G (693)	AGAGATCCCTACACCAGAGAC	GGTGGTGTTGTTTCCGTGCAA
16S V3-V4	ACTCCTACGGGAGGCAGCA	GGACTACHVGGGTWTCTAAT

Supplementary Video. S1 Swimming video of rainbow trout after SB injection with DMEM. Sixteen rainbow trout (~5g) were randomly divided into two groups. One group of fish were SB injected with 50 μL DMEM (SB injection group), while the other fish group where not injected (naïve fish group). After injection, both groups were held in two different 28 L glass tanks (40 cm × 20 cm × 35 cm) at 16 °C for 1 day, respectively. Thereafter, each group fish were imaged for a 1 h period using a Canon 750D video camera.
